# A Flexible Skin Bionic Thermally Comfortable Wearable for Machine Learning‐Facilitated Ultrasensitive Sensing

**DOI:** 10.1002/advs.202401800

**Published:** 2024-06-25

**Authors:** Pengju Di, Yue Yuan, Mingyue Xiao, Zhishan Xu, Yicong Liu, Chenlin Huang, Guangyuan Xu, Liqun Zhang, Pengbo Wan

**Affiliations:** ^1^ College of Materials Science and Engineering State Key Laboratory of Organic‐Inorganic Composites Beijing University of Chemical Technology Beijing 100029 China; ^2^ School of Artificial Intelligence Beijing University of Posts and Telecommunications Beijing 100876 China

**Keywords:** advanced thermal management, flexible electronics, machine learning‐facilitated human‐interactive sensing, MXene, skin bionic microstructure

## Abstract

Tremendous popularity is observed for multifunctional flexible electronics with appealing applications in intelligent electronic skins, human–machine interfaces, and healthcare sensing. However, the reported sensing electronics, mostly can hardly provide ultrasensitive sensing sensitivity, wider sensing range, and robust cycling stability simultaneously, and are limited of efficient heat conduction out from the contacted skin interface after wearing flexible electronics on human skin to satisfy thermal comfort of human skin. Inspired from the ultrasensitive tactile perception microstructure (epidermis/spinosum/signal transmission) of human skin, a flexible comfortably wearable ultrasensitive electronics is hereby prepared from thermal conductive boron nitride nanosheets‐incorporated polyurethane elastomer matrix with MXene nanosheets‐coated surface microdomes as epidermis/spinosum layers assembled with interdigitated electrode as sensing signal transmission layer. It demonstrates appealing sensing performance with ultrasensitive sensitivity (≈288.95 kPa^−1^), up to 300 kPa sensing range, and up to 20 000 sensing cycles from obvious contact area variation between microdome microstructures and the contact electrode under external compression. Furthermore, the bioinspired electronics present advanced thermal management by timely efficient thermal dissipation out from the contacted skin surface to meet human skin thermal comfort with the incorporated thermal conductive boron nitride nanosheets. Thus, it is vitally promising in wearable artificial electronic skins, intelligent human‐interactive sensing, and personal health management.

## Graphical abstract

A flexible skin bionic thermally comfortable wearable is prepared for machine learning‐facilitated ultrasensitive sensing from thermal conductive boron nitride nanosheets‐incorporated polyurethane elastomer substrate with MXene‐coated surface microdomes as epidermis/spinosum layers assembled with contact electrode, featuring ultrahigh sensitivity (up to 288.95 kPa^−1^), wide sensing range, excellent cycling stability, and outstanding thermal management for wearable healthcare monitoring and intelligent human–machine interaction.

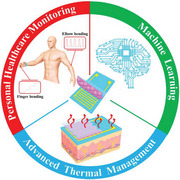

## 1 Introduction

Multifunctional wearables have drawn significant research attention due to their promising in human healthcare monitoring,^[^ ^1^, ^2^ ^]^ smart disease diagnostic sensing and medical treatment,^[^ ^3^, ^4^ ^]^ electronic skins,^[^ ^5^, ^6^ ^]^ and human–machine interface.^[^ ^7^, ^8^ ^]^ Until now, many types of wearable electronic sensors based on different sensing mechanisms, including piezoresistivity, piezoelectricity, capacitance, and triboelectricity, have been developed.^[^ ^9^, ^10^, ^11^ ^]^ In contrast to the other types of sensors, the piezoresistive sensors are drawing tremendous interest in flexible electronics due to their high sensitivity, low energy consumption, and easy fabrication technique. Although numerous prominent research on wearable sensing devices have recently been reported,^[^ ^12^, ^13^ ^]^ it remains a critical challenge to simultaneously achieve high sensitivity and broad working range to detect full‐scale human movements. For instance, a medical pressure sensor, constructed from a MXene‐coated silk fibroin nanofiber membrane and a MXene ink‐coated interdigitated electrode, presented a high sensitivity in a relatively narrow sensing range (up to 39.3 kPa).^[^ ^14^ ^]^ Therefore, it is still urgently desired to develop wearable electronic sensors with high sensitivity, wide sensing range, and reliable cycling stability simultaneously for human–machine interface, personal healthcare monitoring, and smart medical diagnosis.

Human skin is featured with highly sensitive tactile perception capability mainly from the spinosum microstructure with a high specific surface area for amplifying sensing signal and sensitivity in human skin microstructures of protective epidermis, dermis, and nerve conduction layer. Inspired by the highly sensitive tactile perception microstructures of human skin, it is highly desirable to design spinosum‐like microstructures‐contained sensing layer to effectively increase the specific surface area to deposit more conducting sensing materials and to efficiently improve the contact area change between the sensing layers and the electrodes under external pressure, resulting in excellent sensing performance. Nowadays, MXene, metal nanowire, and liquid metal are popular materials candidates to be employed as conducting sensing materials layer in wearable electronics.^[^ ^15^, ^16^, ^17^, ^18^, ^19^, ^20^, ^21^, ^22^ ^]^ In particular, MXene, as a 2D nanomaterial with high conductivity, large specific surface area, excellent mechanical strength, and flexibility, has been versatilely employed in energy storage and harvesting,^[^ ^23^, ^24^ ^]^ electromagnetic interference shielding,^[^ ^25^, ^26^ ^]^ and sensors.^[^ ^27^, ^28^ ^]^ It is worth noting that, MXene, featuring the integrated high conductivity and hydrophilicity, has been considered as ideal nanomaterials to be prepared as active sensing layers of high‐performance sensing electronics.^[^ ^29^ ^]^ Therefore, it is highly desired to develop the skin‐inspired MXene‐based spinosum‐like microstructures‐contained sensing layer in the assembled flexible sensor with superior sensing performance for personalized healthcare detection, next‐generation e‐skins, and wearable human–machine interface.

Furthermore, human core temperature has to be maintained in a narrow range (the comfort zone for human skin is between ≈30 and ≈34 °C^[^ ^30^ ^]^) to ensure the optimal function of the cells and maintain homeostasis. Thus, personal thermal management is highly helpful to control the temperature to maintain the human body with thermal comfort.^[^ ^31^, ^32^, ^33^ ^]^ Recently, related progress in personal thermal management to control the temperature to maintain the human body with thermal comfort, was demonstrated, such as stretchable thermoelectric devices,^[^ ^34^, ^35^, ^36^ ^]^ stretchable heaters^[^ ^37^, ^38^, ^39^ ^]^ and radiative cooling.^[^ ^40^, ^41^ ^]^ The wearable sensing electronics need to be directly attached onto human skin for personal human motion detection, and the corresponding increased temperature and the accumulated heat on the contact interface between flexible sensor and human skin, may evoke discomfort of wearing and sensation because of the poor heat dissipation ability of the mostly reported flexible sensors. In addition, more microorganisms may breed on human skin in the warmer and humid conditions, which may not be good for human health.^[^ ^42^ ^]^ Thus, highly efficient heat dissipation is vitally important to be introduced into the flexible electronics for reducing the corresponding increased temperature and dissipating the accumulated heat on the contact interface between the flexible sensor and human skin for potential comfortable healthy long‐term wearing.

Heating plays an integral role in maintaining human thermal comfort, and the heat generated by wearable electronic devices is anticipated to be efficiently transferred to the human skin for further heating related human body and subsequently thermal therapy.^[^ ^43^, ^44^, ^45^ ^]^ Specifically, photothermal therapy could relieve pain, reduce stiffness, and improve blood circulation, which could be used for arthritis, tenosynovitis, and tennis elbow treatment. Nevertheless, it remains a formidable challenge to realize highly efficient photothermal therapy after healthcare monitoring and medical diagnostic sensing. However, the mostly reported flexible photothermal‐therapy electronics comprised of polymer matrix are short of efficient thermal conducting capability and rapid thermal conducting rate to transfer the generated heat from near‐infrared (NIR) light irradiation to human skins, which may affect the therapeutic effect. The rapidly heating rates could be endowed to the electronics from MXene nanosheets featured with higher efficiency of photothermal conversion, which is promising to be integrated into the highly efficient photothermal electronics.^[^ ^46^ ^]^ Therefore, developing multifunctional wearables featuring superior sensing capability, advanced thermal management, and efficient photothermal therapeutic treatment simultaneously, is strongly desired for great potential in intelligent electronic skins, personal health diagnostic sensing, personalized health management, and medical treatment.

**Scheme 1:**
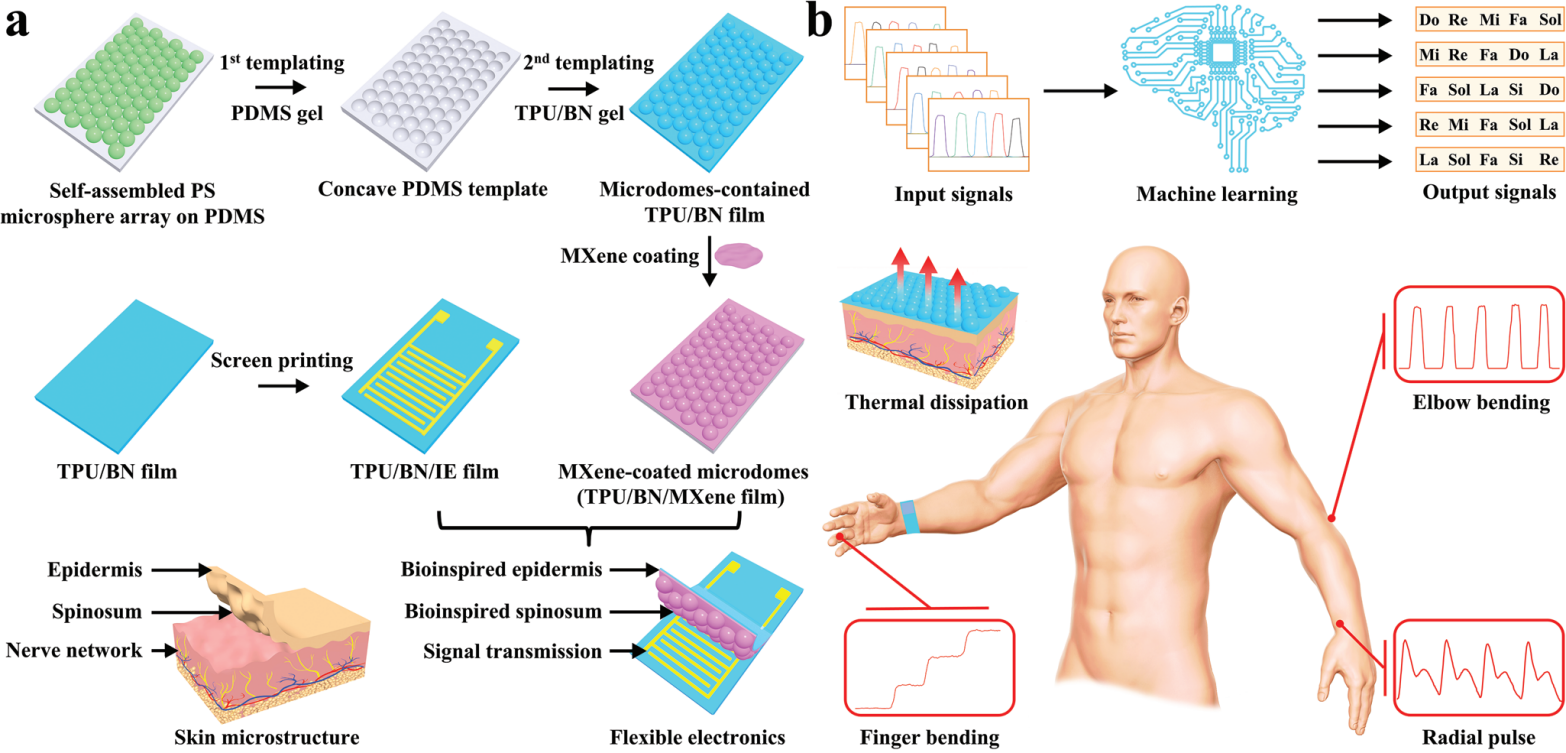
a) Schematic diagram of the fabrication process of a multifunctional flexible wearable electronics by face‐to‐face assembling the thermal conductive boron nitride nanosheets‐incorporated polyurethane elastomer substrate (TPU/BN) with MXene‐coated microdome microstructures (TPU/BN/MXene) and the interdigitated electrode‐coated boron nitride nanosheets‐incorporated polyurethane substrate (TPU/BN/IE) for b) wearable healthcare sensing, machine learning‐enabled human–machine interaction, and TPU/BN film‐facilitated advanced thermal conduction and management of heat out from the contacted human skin surface to reduce the increasing temperature and the accumulated heat.

Herein, we develop a bioinspired flexible wearable multifunctional electronics with superior sensing performance, advanced thermal conduction and management, and reliable photothermal therapy. Bioinspired from human skin with highly sensitive sensing microstructure, a multifunctional flexible sensing electronics is obtained from the thermal conductive boron nitride nanosheets‐incorporated polyurethane elastomer substrate (TPU/BN) with MXene‐coated surface microdome microstructures (TPU/BN/MXene) as epidermis layer/spinosum layer with the assembly of the interdigitated electrode‐coated boron nitride nanosheets‐incorporated polyurethane elastomer (TPU/BN/IE) film as sensing signal transmission layer (**Scheme** **1**). Due to the initial limited contact area and higher contact resistance, and the obvious change of contact area and contact resistance between the microdome structures and the contacted interdigitated electrode during compression, the assembled sensing electronics exhibits higher sensing sensitivity (up to 288.95 kPa^−1^), excellent cycling stability over 20 000 cycles, and broad sensing range (up to 300 kPa), which could be used to sensitively detect various human motions, including pulse, occlusion, and finger bending. Furthermore, the obtained multifunctional electronics, featuring advanced thermal conduction and management, could efficiently dissipate the accumulated heat out timely to maintain the human body in thermal comfort. Besides, the multifunctional electronics presents excellent photothermal ability with the reliable thermal conducting capability from the incorporated thermal conductive boron nitride nanosheets, which could function as a wearable heater, providing instantly enhanced photothermal therapy after wearable healthcare monitoring. Therefore, the multifunctional wearable electronic device with excellent sensing performance, outstanding thermal management, and reliable photothermal effect, has promising potential in wearable healthcare monitoring, intelligent electronic skins, and smart disease diagnosis and treatment.

## 2 Results and Discussion

**Figure 1:**
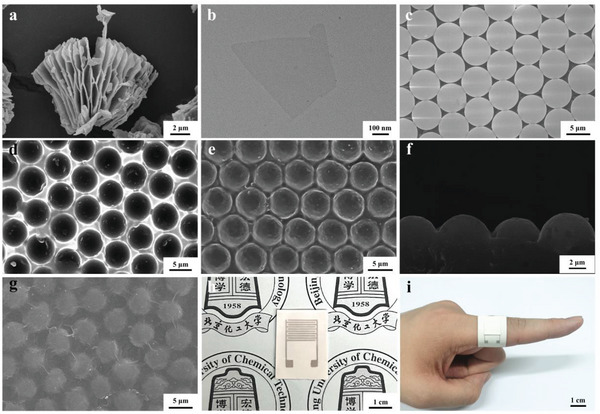
a) SEM image of the unexfoliated MXene. b) TEM image of the MXene nanosheet. c) SEM image of the self‐assembled PS microsphere array. d) SEM image of the negative PDMS template with surface concave microstructure. e) SEM image of the TPU/BN composite with convex microdomes. f) The cross‐sectional SEM image of the TPU/BN composite with convex microdomes. g) SEM image of the TPU/BN/MXene composite with MXene nanosheets‐coated convex microdomes. Photographs of h) the interdigitated electrode‐coated boron nitride nanosheets‐incorporated polyurethane elastomer (TPU/BN/IE) film and i) the assembled flexible sensing electronics attached onto the human finger.

The multifunctional flexible wearable sensing electronics was assembled from a MXene nanosheets‐coated surface microdomes‐contained TPU/BN/MXene film with an interdigitated electrode‐coated TPU/BN/IE film. First, MXene nanosheet was produced by selective etching the Al layer from Ti_3_AlC_2_ precursor with HCl/LiF, and by further sonication and centrifugation (Figure S1, Supporting Information). **Figure** **1**a displays the unexfoliated MXene featuring an accordion‐like structure. Figure 1b and S2 (Supporting Information) showed the transmission electron microscope (TEM) image and the scanning electron microscope (SEM) image of the MXene nanosheet respectively. Furthermore, as illustrated in Figure S3 and S4 (Supporting Information), the leftward shifted (002) peak and the weakened (104) peak in X‐ray diffraction (XRD) patterns confirmed the successful synthesis of the MXene nanosheet. And the X‐ray photoelectron spectroscopy (XPS) revealed the existence of Ti, C, O, and F elements in MXene nanosheets (Figure S5, Supporting Information), indicating the presence of the surface‐terminated groups (─F, ─OH, ═O, etc.). The ultrathin BN with a lateral size of ≈100 nm could be observed in Figure S6 (Supporting Information), and the B and N elements were observed to be the main elements in BN, as shown in the XPS spectra (Figure S7, Supporting Information).

Figure 1c demonstrated a closely and uniformly arranged hexagonal monolayer array microstructure of the self‐assembled polystyrene (PS) microspheres (diameter at ≈5 µm) on polydimethylsiloxane (PDMS) substrate obtained by the rubbing process. Then, the negative concave PDMS template was fabricated by coating PDMS curing gel on the self‐assembled PS microspheres‐based hexagonal monolayer array microstructure surface and by subsequently sacrificing PS microspheres‐based hexagonal monolayer array microstructure after curing of PDMS gel (Figure 1d). Subsequently, the thermal conductive boron nitride nanosheets‐incorporated polyurethane elastomer substrate (TPU/BN) with surface convex microdome microstructures were obtained by coating TPU/BN‐contained *N*, *N*‐Dimethylformamide (DMF) solution, curing and peeling off (Figure 1e). Figure 1f shows the cross‐sectional SEM image of the surface convex microdome microstructures‐contained TPU/BN film with the microdome height at ≈2.5 µm. After coating MXene nanosheets on the surface convex microdome microstructures, the MXene‐coated surface microdome microstructures‐contained TPU/BN/MXene film was obtained (Figure 1g). As shown in Figure S4 (Supporting Information), the characteristic peaks of TPU film, BN, and MXene nanosheets could be observed in the TPU/BN/MXene composite at 6.62° (the (002) crystal plane of MXene), 19.74° (the amorphous region of TPU), and 26.9° (the (002) basal plane of BN), respectively, demonstrating the successful construction of the TPU/BN/MXene composite. The interdigitated electrode patterns were constructed on the flat TPU/BN film through screen‐printing conductive silver adhesives, and the dimensions and the photograph of the TPU/BN/IE film were shown in Figure 1h and Figure S8 (Supporting Information). Finally, the flexible wearable electronics was successfully assembled with robust flexibility, as shown in Figure 1i.

**Figure 2:**
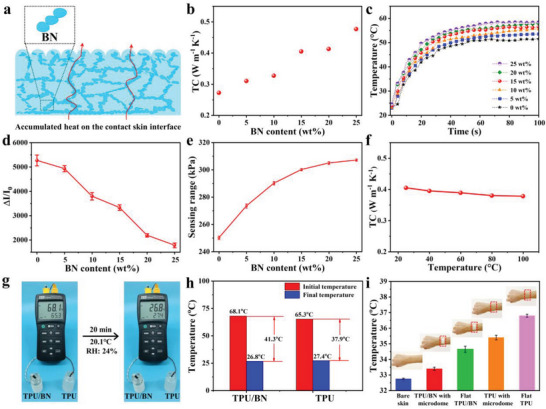
a) Schematic diagram of the thermal conduction of the TPU/BN composite with microdomes surface microstructure. b) TC of the TPU/BN composites with microdomes surface microstructure as a function of BN mass fraction. c) The corresponding upper surface real‐time temperature of the TPU/BN composites with microdomes surface microstructure under different BN loading placed on the hot plate (≈60 °C), and recorded with the IR camera. d) The sensing responses of the assembled sensors with different BN contents at 50 kPa. e) The maximum sensing range of the assembled sensors as a function of BN mass fraction. f) TC of the TPU/BN composite with microdomes surface microstructure under 15 wt.% BN loading at different environmental temperatures. g) The thermal diffusion experiment of the microdomes surface microstructure‐contained TPU/BN composite and a pure TPU film. The recorded real‐time temperature of the hot water in the bottles with the coating of the microdomes surface microstructure‐contained TPU/BN composite on its outer bottle wall and with the coating of the pure TPU film on the outer bottle wall respectively. h) The recorded temperatures of the hot water in the bottles coated with the corresponding films on the outer bottle walls respectively. i) The skin surface temperatures under various skin coating: bare skin, the microdomes surface microstructure‐contained TPU/BN composite, the flat TPU/BN composite, the microdomes surface microstructure‐contained pure TPU film, and the flat pure TPU film coating.

The mostly reported flexible electronics composed of polymer matrix are lack of efficient thermal conduction and management capability to satisfy the thermal comfort in human skins for long‐period comfortable wearing. As shown in **Figure** **2**a, the incorporated thermal conductive BN network in the TPU matrix was helpful to facilitate the highly efficient thermal conduction performance. As demonstrated in Figure 2b, the thermal conductivity (TC) of the TPU/BN composites with microdomes surface microstructure increased along with the gradually increasing BN content, and the TC of the TPU/BN composite with microdomes surface microstructure under 15 wt.% BN content was 0.405 W m^−1^ K^−1^, more than 149% enhancement compared with that of pure TPU film. The thermal conducting BN material in the TPU/BN composite overlapped and interconnected with each other in the TPU matrix to form the thermal conducting paths. The TPU/BN composites with microdomes surface microstructure under different BN loading were placed on the hot plate (≈60 °C), and the corresponding upper surface temperature of the composite was recorded with the IR camera (Figure 2c). The upper surface temperature of the composite with 15 wt.% BN loading could reach to ≈56 °C within 60 s, indicating that the introduction of BN could effectively improve the TC of TPU/BN composite with microdomes surface microstructure for promising thermal management.

The sensing performances of the sensors assembled from MXene nanosheets‐covered surface microdomes‐contained TPU/BN/MXene film with different BN contents and an interdigitated electrode film were shown in Figure 2d. The tensile stress–strain curves of the TPU elastomers with various BN contents were displayed in Figure S9 (Supporting Information). With the increasing BN contents, the elastic moduli of MXene nanosheets‐coated surface microdomes‐contained TPU/BN/MXene film increased and their deformation decreased under the same pressure loading, resulting in the smaller contact area change between MXene nanosheets‐coated surface microdomes‐contained TPU/BN/MXene film and an interdigitated electrode film, the decreased contact resistance change, the lower sensing response and sensing sensitivity (Figures S9, S10, Supporting Information). The sensing capabilities of the flexible electronic sensors made from TPU elastomers incorporated with different BN contents and the corresponding variable elastic moduli (TPU (≈3.9 MPa), TPU/8%BN (≈6.8 MPa), TPU/15%BN (≈13.6 MPa), TPU/20%BN (≈18.4 MPa), and TPU/25%BN (≈24.9 MPa)) were shown in Figure S10 (Supporting Information) respectively. Because of the increased moduli of the TPU/BN/MXene films with the increasing BN contents, the corresponding TPU/BN/MXene films could bear larger pressure loading until deformation saturation, eventually generating a broader detection range of the flexible electronics (Figure 2e). It can be seen that the flexible electronic sensors prepared from TPU/15%BN elastomer matrix with an elastic modulus of ≈13.6 MPa had both relatively higher sensing sensitivity and wider sensing range, as shown in Figure S10 (Supporting Information) and Figure 2e. This may be related to the different degrees of compressive deformation and the different relative contact area changes of the flexible sensors prepared from elastomers with different moduli under external pressure. The sensors prepared from the TPU elastomers with lower BN loading and relatively lower elastic moduli, showed a relatively higher contact area change and quickly reached compressive deformation saturation under relatively lower external pressure loading, resulting in a relatively higher sensing sensitivity and a narrow sensing range. The sensors prepared from the TPU elastomers with relatively higher BN addition had relatively limited compression deformation under the same external pressure, resulting in a relatively smaller change of the contact area, a relatively lower sensing sensitivity, and a relatively wider sensing range for withstanding a large external pressure loading. Therefore, the MXene nanosheets‐coated surface microdomes‐contained TPU/BN/MXene film with 15 wt.% BN loading was optimally selected for advanced thermal conduction and management, and assembled for flexible electronics with optimized sensing performance (relatively higher sensing sensitivity and wider sensing range simultaneously) employed for further detailed promising sensing applications.

Figure 2f demonstrates the TC of the TPU/BN composite with microdomes surface microstructure under 15 wt.% BN loading at different environmental temperatures, indicating the stable heat‐conducting property of the TPU/BN composite with microdomes surface microstructure, which could meet the requirement of thermal comfort by efficiently stably dissipating the accumulated heat. As shown in Figure 2g,h, the temperature of the hot water (68.1 °C) in the bottle with the coating of the TPU/BN composite with microdomes surface microstructure on its outer bottle wall decreased from 68.1 to 26.8 °C for 20 min at the environmental temperature of 20.1 °C, while the temperature of the hot water (65.3 °C) in the bottle with the coating of the pure TPU film on the outer bottle wall decreased from 65.3 to 27.4 °C at the same period. The obvious hot water decrease (41.3 °C) for the bottle with the coating of the TPU/BN composite with microdomes surface microstructure on its outer bottle wall could be observed, in comparison with that (37.9 °C) for the bottle with the coating of the pure TPU film on the outer bottle wall, which is mainly attributing to excellent thermal conduction of the coated TPU/BN composite with microdomes surface microstructure. The accumulated heat on the contact interface between flexible sensor and human skin from human metabolism is usually radiated through radiation, conduction, convection, and evaporation to the outer environment.^[^ ^41^, ^47^, ^48^, ^49^ ^]^ Figure 2i shows the skin surface temperatures under various skin coating conditions (bare skin at ≈32.75 °C, TPU/BN composite with microdomes surface microstructure at ≈33.40 °C, flat TPU/BN film at ≈34.65 °C, pure TPU film with microdomes surface microstructure at ≈35.40 °C, and flat pure TPU film at ≈36.80 °C). Compared with the pure TPU film with microdomes surface microstructure, the TPU/BN composite film with microdomes surface microstructure possessed a lower surface temperature because of the incorporated thermal conductive BN network in TPU matrix (Figure S11, Supporting Information) to facilitate the highly efficient thermal conduction performance. Compared with the flat TPU/BN composite film, the TPU/BN composite film with microdomes surface microstructure possessed better heat dissipation capacity, which is due to the existence of microdome microstructures on the surface of the film to increase the contact area between the film and the outer air and environment, resulting in enhanced heat dissipation of radiation and convective. As shown in Figure S12 (Supporting Information), compared to the L929 cells cultured in the blank groups, the L929 cells cultured in the extract substrate of the TPU/BN composite for 24, 48, and 72 h showed a normal fusiform morphology and similar cell density. Besides, the relative growth rate value of the L929 cells cultured in the extract substrate of the TPU/BN composite was comparable to that of the blank group, revealing that the TPU/BN composite contacted with skin was non‐toxic and biocompatible. Therefore, the as‐prepared TPU/BN composite with microdomes surface microstructure featuring outstanding heat dissipation capability, excellent thermal conduction performance compared with other kinds of literature (Figure S13, Supporting Information),^[^ ^50^, ^51^, ^52^, ^53^ ^]^ and reliable biocompatibility, holds great potential in flexible wearable electronic devices and smart electronic skins with fascinating advanced thermal conduction and highly efficient thermal management.

The sensing responses of the flexible wearable electronic sensors assembled from MXene nanosheets‐covered surface microdomes‐included TPU/BN/MXene film and an interdigitated electrode‐coated TPU/BN/IE film were demonstrated in Figure S14 (Supporting Information). Along with the increasing MXene coating, a uniform and complete conducting network layer was formed on the surface of the microdomes‐contained TPU/BN/MXene film gradually, resulting in the increasing conductivity of the MXene nanosheets‐coated surface microdomes‐contained TPU/BN/MXene film and the higher sensing response of the flexible electronics. The excessive MXene nanosheets were coated on the surface microdomes of the TPU/BN/MXene film, leading to the covered surface rough microstructures, the decreased surface specific area, the reduced contact area change, the decreasing contact resistance change, and the lower sensing response. Consequently, the MXene nanosheets‐coated surface microdomes‐contained TPU/BN/MXene film with 0.06 mg MXene nanosheets coating could be the optimized candidate to be assembled in the sensitive flexible electronics.

**Figure 3:**
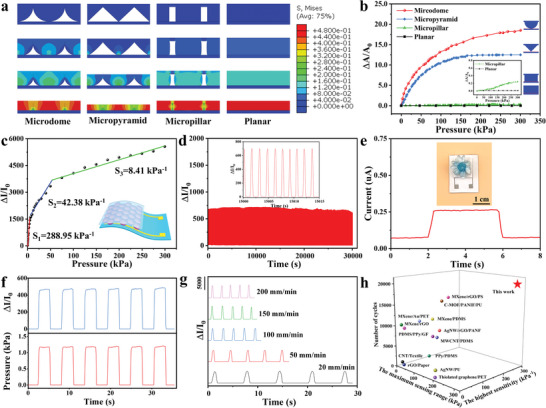
a) The finite‐element simulation of the stress distribution for the array microstructures with different morphologies (microdome, micropyramid, micropillar, and planar structure) with the contact electrodes under the pressures of 0, 15, 70, and 300 kPa respectively. b) The relative contact area change (Δ*A*/*A*_0_) between the array microstructures with different morphologies (microdome, micropyramid, micropillar, and planar structure) and the contacted electrodes under different pressures calculated by finite‐element simulation respectively. c) The sensing sensitivity of the flexible electronics. d) The cycling stability of the flexible electronics under the pressure of 2 kPa. Inset: the detailed cycling stability under the pressure of 2 kPa. e) The experimental detection limit of the flexible electronics to a plastic paper flower. f) The output current sensing response and the input pressure waves at 1.2 kPa as a function of time. g) The sensing responses of the flexible electronics under different compression rates. h) Comparison of the flexible electronics with other reported sensors in terms of the highest sensitivity, the maximum working range, and the cycle number.^[^ ^13^, ^27^, ^54^, ^55^, ^56^, ^57^, ^58^, ^59^, ^60^, ^61^, ^62^, ^63^, ^64^ ^]^

**Figure** **3**a demonstrates the finite‐element simulation (FEA) of the stress distribution within the array microstructures with different morphologies (microdome, micropyramid, and micropillar) with the contact electrodes under the pressures of 0, 15, 70, and 300 kPa respectively. It could be seen that under the same applied pressure loading, the three microstructures experienced different stress distributions due to their different geometries (Figure 3a). As shown in Figure 3b, the relative contact area change (Δ*A*/*A*_0_) between the microstructures with different morphologies (microdome, micropyramid, micropillar, and planar structure) and the contacted electrodes under different pressures calculated by finite‐element simulation, could be observed. Under an external pressure loading, the stress distribution was apparently nonuniform for the microdome and micropyramid, and the local stress was concentrated near the contacting tips due to the smaller contact area, which results in higher mechanical deformations and rapid obvious changes of the relative contact area (Figure 3b). For the micropillar, a relatively homogeneous local stress distribution could be observed under external pressure, resulting in an inconspicuous change of the relative contact area (Figure 3b). The significantly inconspicuous change of the relative contact area could be observed for planar structure than that for other microstructures. Along with the further increasing external pressure loading, the deformation of the microstructures tended to be gradually saturated, resulting in a relatively limited contact area change under the larger pressure loading (Figure 3b). Compared with the other microstructures, the mircodome exhibited relatively higher contact area change (Δ*A*/*A*_0_) under the same pressure loading. When a smaller external pressure was applied (*P* = 15 kPa in Figure 3a), obvious stress concentration and significant mechanical deformation of the microdome array microstructures could be observed, leading to a tremendously increased contact area change (Figure 3b). Therefore, the highest sensing sensitivity under a smaller applied external pressure (288.95 kPa^−1^ in Figure 3c) could be obtained for the tremendously increased contact area change and the limited initial contact area between the microdome array microstructures and the corresponding contact electrode. With a gradually increased pressure loading (*P *= 70 kPa in Figure 3a), a relatively obvious deformation of the microdome array microstructures could be observed, resulting in a relatively higher contact area change (Figure 3b), and finally a relatively higher sensing sensitivity of the flexible electronics (42.38 kPa ^−1^ in Figure 3c). Along with an obviously increased pressure loading (*P* = 300 kPa in Figure 3a), the mechanical deformation of the microdome array microstructures gradually reached saturation, resulting in a relatively limited contact area change (Figure 3b), and finally a relatively high sensing sensitivity (8.41 kPa^−1^ in Figure 3c).

As shown in Figure S15 (Supporting Information), the sensing responses of the flexible electronics under different pressures (up to 300 kPa) could be facilely obtained. The sensing sensitivity (*S*) is a vital parameter for evaluating the sensing performance of flexible electronic sensors, which is defined as^[^ ^46^ ^]^

(1) $$S=\frac{\delta \left(\frac{\Delta I}{I_{0}}\right)}{\delta P}=\frac{\delta \left(\frac{R_{0}}{R_{t}}-1\right)}{\delta P}\approx \frac{\delta \left(\frac{R_{contact,0}}{R_{contact,t}}-1\right)}{\delta P}=\frac{\delta \left(\frac{\Delta A}{A_{0}}\right)}{\delta P}$$

where ∆*I* stands for the change in current, *I*_0_ represents the initial current of the sensor and *P* is the pressure applied to the sensor. *R*_0_ represents the initial resistance of the sensor, *R*_t_ is the real‐time resistance of the sensor under external pressure, *R*_contact,0_ is the initial contact resistance between the microdome structures and the contacted interdigitated electrode, and *R*_contact,t_ stands for the real‐time contact resistance between the microdome structures and the contacted interdigitated electrode under external pressure. ∆*A* stands for the real‐time change of the contact area between the microdome structures and the contacted interdigitated electrode, *A*_0_ is the initial contact area between the microdome structures and the contacted interdigitated electrode. Due to the limited initial contact area *A*_0_ and the higher initial contact resistance *R*_contact,0_, and the apparent variation of the contact area ∆*A* and the lower contact resistance *R*_contact,t_ between the microdome structures and the contacted interdigitated electrode during compression, the superior highly sensitively sensing performance featuring broader sensing range (up to 300 kPa) and highest sensing sensitivity (up to 288.95 kPa^−1^), could be obtained (Figure 3c). The flexible electronics (Figure S16a, Supporting Information) assembled from MXene nanosheets‐coated flat TPU/BN/MXene film and an interdigitated electrode‐coated TPU/BN/IE film, possessed the relatively better original contact area (*A*_0_), the relatively inferior original contact resistance (*R*_contact,0_), and the relatively inferior contact area change (∆*A*) at the contact interface between the MXene nanosheets‐coated flat TPU/BN/MXene film and the interdigitated electrode‐coated TPU/BN/IE film during compression, leading to a relatively lower sensing response and a relatively smaller sensitivity. The flexible sensing electronics (Figure S16b, Supporting Information) assembled from MXene nanosheets‐coated surface microdomes‐contained TPU/BN/MXene film and the two conductive stripes‐contained electrode‐coated TPU/BN film possessed the limited change of the contact area ∆*A* between the microdome structures and the two conductive stripes‐contained electrode‐coated TPU/BN film, leading to a relatively smaller sensing response and a relatively lower sensing sensitivity. The flexible sensing electronics (Figure S16c, Supporting Information) assembled from MXene nanosheets‐coated surface microdomes‐contained TPU/BN/MXene film and the conductive planar electrode‐coated TPU/BN film possessed the relatively higher initial contact area *A*_0_, leading to a relatively inferior sensitivity. The flexible sensing electronics (Figure S16d, Supporting Information) assembled from two MXene nanosheets‐coated surface microdomes‐contained TPU/BN/MXene films face‐to‐face, possessed the relatively higher original contact area *A*_0_ between two MXene nanosheets‐coated surface microdomes‐contained TPU/BN/MXene films, mainly leading to a relatively smaller sensing response and a relatively lower sensing sensitivity. Therefore, compared to the above as‐prepared various flexible electronics, the flexible electronics assembled from a MXene nanosheets‐coated surface microdomes‐contained TPU/BN/MXene film with an interdigitated electrode‐coated TPU/BN/IE film, exhibited the highest sensitivity (Figure S16e,f, Supporting Information), indicating the reliable potential for ultrasensitive human motion detection and clinic diagnosis.

Figure 3d demonstrates the cycling stability of the flexible electronics for ≈20 000 compressive loading–unloading cycles, implying remarkable stability of the flexible electronics for long‐period personal health monitoring. As shown in Figure 3e, the flexible electronics could sensitively detect minor pressure (0.95 Pa) generated from a plastic paper flower loading (9.5 mg). Furthermore, Figure 3f shows the output current sensing response and the input pressure waves at 1.2 kPa as a function of time, illustrating no obvious hysteresis, the fast response time, and the reliable sensing capability of the flexible electronics. The response time and the recovery time were further measured to be ≈17 and ≈26 ms respectively, assuring the great potential for real‐time human motion monitoring in flexible electronics (Figure S17, Supporting Information). As shown in Figure S18 (Supporting Information), the current–voltage (*I*–*V*) curves of the flexible electronics under different external pressures, presented the linear relation, indicating the ohmic contact behavior between TPU/BN/MXene film with the MXene nanosheets‐covered surface microdomes and the contacted interdigitated electrode. As shown in Figure 3g, the flexible electronics exhibited robust sensing stability under different compression rates. Finally, Figure 3h and Table S1 (Supporting Information) show the sensing performance comparison of this flexible electronics with other reported pressure sensors,^[^ ^13^, ^27^, ^54^, ^55^, ^56^, ^57^, ^58^, ^59^, ^60^, ^61^, ^62^, ^63^, ^64^ ^]^ and it is evident that this flexible electronics had the highest sensing sensitivity (up to 288.95 kPa^−1^), a wider detection range (up to 300 kPa), and excellent cycle stability (20 000 cycles).

**Figure 4:**
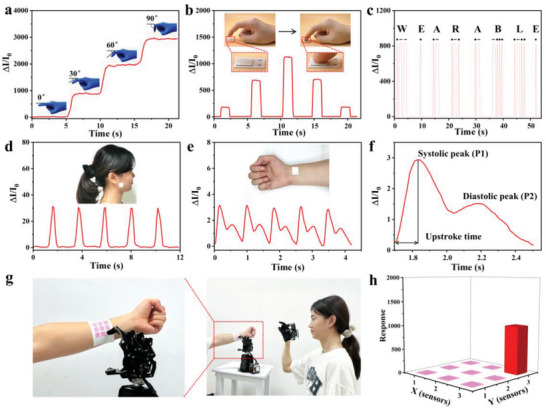
a) Sensing performances of the flexible electronics to finger bending (Inset: photograph of the flexible electronics attached onto the finger joint under different finger bending angles). b) Sensing performances of the flexible electronics to different pressures produced by finger pressing (Inset: Photograph of finger tapping to the flexible electronics). c) Sensing performance of the flexible electronics to Morse Code “wearable” generated by finger knocking with different rhythms. d) Sensing performances of the flexible electronics to occlusion (Inset: photograph of the flexible electronics attached to human cheek). e,f) Sensing performance of the flexible electronics to the wrist pulse (Inset: photograph of the flexible electronics adhered to wrist pulse position). g) Photograph of the electronic skin assembled from the flexible electronics with 3 × 3 pixels attached on the volunteer's forearm. h) The corresponding pressure distribution recorded by the assembled electronic skin attached on the human skin by the manipulator's finger pressing under the wireless control of a volunteer with a wearable wireless somatosensory glove.

The flexible electronics, featuring with high sensitivity, broad working range, excellent cycle stability, and fast response time, is a promising and attractive electronic device for large‐scale human motion detection, tiny human movements monitoring, and clinical diagnosis. As shown in **Figure** **4**a, the flexible electronics was attached to the finger of a volunteer to detect the finger bending, and the sensing responses increased with the increased bending angle, which could be used for real‐time precise and stable detection in rehabilitation exercise. Furthermore, the flexible electronics could clearly detect the different pressures generated from different finger‐tapping pressures, and the sensing responses of the flexible electronics varied along with the gradually variable finger‐pressing pressures (Figure 4b). The Morse code (consisting of two signals “Di·”and “Da─”) for “wearable” could be obtained by the flexible electronics under different rhythms of the finger pressing, which is significant for encrypted information transmission and security communication (Figure 4c).

Furthermore, the flexible electronics could also be used for tiny human motion detection. For example, it could be attached to the cheek of a volunteer for occlusion sensing (Figure 4d). Meanwhile, the clear and relatively stable blood pulse signals could be detected in real time when the flexible electronics was attached onto the corresponding artery position of the human wrist^[^ ^65^ ^]^ (Figure 4e). As shown in Figure 4f, the heart rate of the volunteers (≈75 beats min^−1^) could be obtained from the pulse wave, which is consistent with the physiological heart rate range of healthy adults (60–100 beats min^−1^). Furthermore, the characteristic peaks (contraction peak (P1) and reflection wave (P2)) could be observed, and the radial enhancement index (*AI*_r_ = P2/P1) could be calculated, which is one of the important reference indicators to reflect arteriosclerosis and vascular aging for predicting potential cardiovascular diseases risk. The radial enhancement index could be calculated to be ≈0.52, which is consistent with the value reported in the kinds of literature for 28‐year‐old healthy men, proving that the volunteer is in good cardiovascular health. In addition, the upstroke time is a vital parameter in the evaluation of the artery condition, which was defined as the ascending time of the systolic stage, and could be measured to be ≈165 ms, which is within the normal range (less than 180 ms).^[^ ^66^, ^67^ ^]^ Therefore, flexible electronics shows great potential in long‐term real‐time cardiovascular status monitoring, medical diagnosis, and personal health management. In addition, flexible electronics could be assembled to intelligent artificial electronic skins for sensing pressure distribution. Figure 4g shows the photographs of the assembled electronic skin attached on the human skin from the manipulator's finger pressing with the wireless control of a volunteer with a wearable wireless somatosensory glove, along with the corresponding pressure distribution in Figure 4h respectively. Therefore, the multifunctional flexible wearable sensing electronics with excellent sensing properties is a promising candidate for smart electronic skins, personal healthcare monitoring, human‐interactive sensing, and smart medical diagnosis.

**Figure 5:**
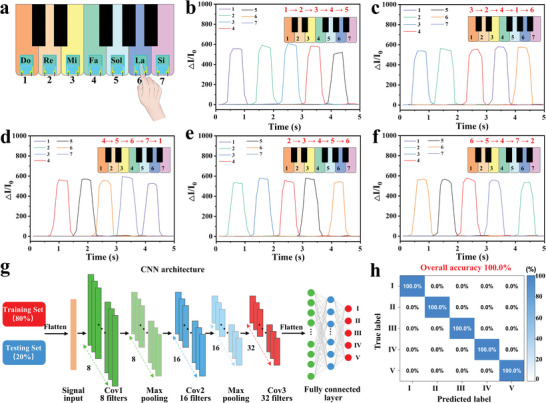
a) Schematic diagram of the pressing of the intelligent piano with sensors‐integrated keyboards to demonstrate different tones. b–f) The sensing responses of the sensors coated on the keyboards of the intelligent piano to five different tones from finger pressing. g) Machine learning algorithm based on CNN for the recognition of different tones: I) 12345, II) 32416, III) 45671, IV) 23456, and V) 65472. h) Confusion matrix results for different tones identification.

In addition, a flexible electronics array was assembled from an intelligent piano with the flexible sensors coated onto the piano keyboards for human finger pressing sensing to different tones from different finger pressing sequences (**Figure** **5**a). As shown in Figure 5b–f, the volunteer played five different tones by different finger pressing sequences, including “Do Re Mi Fa Sol”, “Mi Re Fa Do La”, “Fa So La Si Do”, “Re Mi Fa Sol La”, and “La So Fa Si Re”, the corresponding sensing signals to different tones from finger pressing would be obtained, indicating promising potential in next‐generation human–machine interaction sensing and intelligent human–machine interface. With the development of artificial intelligence, intelligent human‐interactive systems based on human–machine interfaces have become an important potential trend nowadays. A convolutional neural network (CNN) model based on the 1D‐CNN algorithm was designed for classifying and recognizing five different tones along with the corresponding input sensing signals, as shown in Figure 5g. To guarantee the accurate identification of the sensing signals for different tones, the corresponding sensing signals to each tone were tested for 100 times to build the dataset for training and distinguishing each tone. In order to obtain robust training and evaluation, the dataset was randomly divided into two non‐overlapping subsets, with 80% designated for training and 20% for testing. With the increasing training epochs, the classification accuracy was gradually improved up to 100%, while the cross‐entropy loss function gradually decreased (Figure S19, Supporting Information). As shown in Figure 5h, the as‐obtained recognition accuracy could be up to 100%, opening up promising potential in machine learning‐assisted human–machine interaction sensing and intelligent human–machine interface.

MXene, featured with high photothermal conversion efficiency (≈100%),^[^ ^68^ ^]^ endows the prepared MXene nanosheets‐coated surface microdomes‐contained TPU/BN/MXene films with superior photothermal capability for photothermal therapy. Owing to the smaller interfacial barrier resulted from the supramolecular interfacial interaction between MXene nanosheets and the heat‐conductive substrate, the interfacial phonon scattering during the heat transfer process is significantly reduced, leading to an effective heat transfer performance. Besides, TPU/BN/MXene films featuring the highly efficient thermal conducting capability from the incorporated thermal conductive boron nitride nanosheets could transfer the generated heat from the MXene nanosheets under 808 nm NIR light irradiation to human skin surface efficiently, assuring the potential for further enhanced photothermal therapy after personal healthcare monitoring. Figure S20 (Supporting Information) presented the normalized UV–Vis–NIR absorption spectra of surface microdomes‐contained TPU/BN film and MXene nanosheets‐coated surface microdomes‐contained TPU/BN/MXene film, demonstrating a higher absorption in the NIR region of 750–810 nm in Figure S20 (Supporting Information) for MXene nanosheets‐coated surface microdomes‐contained TPU/BN/MXene film from the superior NIR light absorption ability of MXene nanosheets. The temperature‐time curves of MXene nanosheets‐coated surface microdomes‐contained TPU/BN/MXene films under 808 nm NIR light irradiation at variable power densities are shown in Figure S21a (Supporting Information). The temperature of the MXene nanosheets‐coated surface microdomes‐included TPU/BN/MXene film first increased rapidly, reaching a saturated surface temperature within ≈100 s, and quickly dropped to room temperature after turning off the NIR light, indicating its fast and sensitive photothermal ability. The temperature−time curves of MXene nanosheets‐covered surface microdomes‐contained TPU/MXene film and MXene nanosheets‐coated surface microdomes‐contained TPU/BN/MXene film under 808 nm NIR light irradiation at the power density of 0.50 W cm^−2^ was shown in Figure S21b (Supporting Information), presenting a rapid temperature change and higher saturated temperature for MXene nanosheets‐coated surface microdomes‐contained TPU/BN/MXene films from the incorporated superior thermal conductive boron nitride nanosheets in PU matrix, which is a promising candidate for fast and efficient photothermal treatment.

As shown in Figure S21c (Supporting Information), along with the increased power density, the temperature change ∆*T* (the temperature change from saturated temperature to experimental room temperature) of TPU/BN/MXene film with MXene nanosheets‐covered surface microdomes increased linearly. Thus, the surface temperature of TPU/BN/MXene film with MXene nanosheets‐covered surface microdomes could be facilely controlled to meet different temperature requirements by adjusting the NIR light power density. Subsequently, the MXene nanosheets‐coated surface microdomes‐contained TPU/BN/MXene film presented stably cyclic photothermal heating performance under reversibly switching of NIR light irradiation (Figure S21d, Supporting Information), indicating its robust photothermal heating performance. Therefore, the MXene nanosheets‐coated surface microdomes‐contained TPU/BN/MXene film could provide convenient, fast, and stable photothermal heating and on‐demand temperature, which could be employed for further photothermal therapy by boosting the human body's metabolism, increasing blood circulation, and reducing pain.^[^ ^44^, ^46^ ^]^ After wearing the flexible electronics‐integrated elbow band by a volunteer, the elbow bending could be monitored by the flexible electronics for intelligent clinical diagnosis. As shown in Figure S21e (Supporting Information), the volunteer with a painful elbow (such as tennis elbow) may experience pain and stiffness during elbow bending and was unable to bend at a large angle, resulting in a relatively lower sensing response value and unstable sensing signals. The 808 nm NIR light irradiation was performed on the MXene nanosheets‐covered surface microdomes‐contained TPU/BN/MXene film coated on the elbow for highly efficient photothermal treatment of the elbow, to facilitate blood circulation and apparently improve pain and stiffness (Figure S21f, Supporting Information). After efficient photothermal therapy, a relatively stable sensing response could be obtained (Figure S21g, Supporting Information). Therefore, the multifunctional flexible electronics is an excellent candidate to diagnose and treat arthritis, tenosynovitis, and tennis elbow, and could be employed for efficient NIR light‐irradiated photothermal therapy, featuring on‐demand regulation of the required temperature, and fast and sensitive photothermal heating ability.

**Figure 6:**
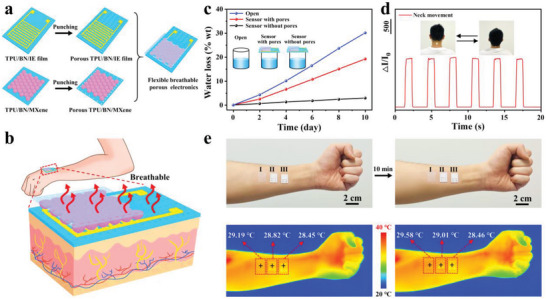
a) Schematic diagram of the preparation process of a flexible breathable porous wearable electronics by face‐to‐face assembling the breathable, porous, thermal conductive boron nitride nanosheets‐incorporated polyurethane elastomer substrate (TPU/BN) with MXene‐coated microdome microstructures (porous TPU/BN/MXene) prepared by punching (punched pore size: diameter at 0.2 mm) with the breathable porous interdigitated electrode‐coated boron nitride nanosheets‐incorporated polyurethane substrate (porous TPU/BN/IE) prepared by punching (punched pore size: diameter at 0.2 mm) b) for thermally comfortable long‐term wearing on human skin. c) The water content loss in bottles filled with water and covered by different films (open, flexible electronics, and flexible breathable porous electronics). d) The sensing performance of the flexible breathable porous wearable electronics to the neck movement. e) Photographs of the pure TPU film (I), the flexible wearable electronics without pores (II), and the flexible breathable porous wearable electronics (III), coated onto human skin respectively, and the corresponding IR thermal images.

Furthermore, the excellent breathability and the further improved thermal dissipation ability of the flexible breathable electronics directly attached onto human skin to ensure long‐term wearable comfortability with robust air/vapor permeability, are highly desired (**Figure** **6**a,b). The breathable, porous, thermal conductive boron nitride nanosheets‐incorporated polyurethane elastomer substrate (TPU/BN) with MXene‐coated microdome microstructures (porous TPU/BN/MXene) prepared by punching (punched pore size: diameter at 0.2 mm) was assembled with the breathable porous interdigitated electrode‐coated boron nitride nanosheets‐incorporated polyurethane substrate (porous TPU/BN/IE) prepared by punching (punched pore size: diameter at 0.2 mm) face‐to‐face to obtain the flexible breathable porous wearable electronics. The breathability was measured by the water content loss in bottles filled with water and covered by different films (open, flexible electronics, and flexible breathable porous electronics) in Figure 6c. It could be seen that the flexible breathable porous wearable electronics exhibited excellent breathability (water loss: 1.9 wt.% per day), which was much higher than that for the flexible wearable electronics without pores (Figure 6c). In addition, the flexible breathable porous wearable electronics demonstrated excellent sensing performance, which showed stable signal monitoring when detecting neck movement (Figure 6d). Figure 6e confirmed that the surface temperature of the flexible breathable porous wearable electronics coated onto human skin was the lowest one compared to that for the pure TPU film and the flexible wearable electronics without pores coated onto human skin respectively after wearing for 10 min by infrared (IR) imaging. Thus, thanks to the excellent TC of boron nitride nanosheets in the TPU/BN matrix and the breathable porous structure in the flexible electronics, the flexible breathable porous wearable electronics possessed excellent breathability and synergistically improved thermal dissipation ability to ensure the wearable comfortability of human skin.

## 3 Conclusion

In this work, bioinspired from tactile perception microstructure of human skin with highly sensitive sensing performance, a multifunctional flexible wearable electronics is assembled from MXene‐coated thermal conductive boron nitride nanosheets‐incorporated polyurethane elastomer substrate with microdome microstructures and the interdigitated electrode‐coated boron nitride nanosheets‐incorporated polyurethane elastomer film face‐to‐face for superior sensing performance, brilliant thermal management, and efficient photothermal therapy. Due to the limited initial contact area and higher contact resistance, and the obvious change of contact area and contact resistance between the microdome structures and the contacted electrode during compression as proved by both experimental and finite‐element simulation results, the resultant flexible electronics presented superior sensing performance including a wide sensing range (up to 300 kPa), high sensitivity (up to 288.95 kPa^−1^), and robust cyclic stability over 20 000 cycles, which could be used for personal healthcare monitoring (such as finger bending, occlusion, and blood pulse), advanced healthy management, and intelligent electronic skins. Machine learning‐assisted human–machine interaction with 100% recognition accuracy was obtained for the collected sensing signals from the flexible sensors coated onto the piano keyboards for human finger pressing sensing to different melodies from related finger pressing. Moreover, the boron nitride nanosheets‐incorporated polyurethane elastomer substrate with microdome microstructures exhibited an enhanced TC because of the incorporated thermal conductive BN network, which can efficiently dissipate the accumulated heat out in time and maintain the human body in thermal comfort. Subsequently, the MXene‐coated thermal conductive boron nitride nanosheets‐incorporated polyurethane elastomer substrate with microdome microstructures presented reliable and effective photothermal heating ability with the reliable thermal conducting capability from the incorporated thermal conducting BN nanosheets, working as an intelligent flexible heater for controlled photothermal therapy of tennis elbow and tenosynovitis by reducing the pain, relieving the stiffness, and promoting the blood flow. Therefore, the multifunctional flexible wearable elastomer materials and electronics with excellent sensing performance, outstanding thermal conduction and management, and reliable photothermal effect, are highly promising for personal health management, smart electronic skins, and intelligent disease diagnosis and treatment.

## 4 Experimental Section

### Materials

Ti_3_AlC_2_ MAX phase was procured from 11 Technology Co. Ltd. Lithium fluoride (LiF) was obtained from Alfa Aesar. Polystyrene (PS) microspheres dispersion was procured from Aladdin Chemical Co. Polydimethylsiloxane (PDMS) prepolymer (Sylgard 184) and curing agent were obtained from Dow Corning. Boron nitride nanosheets (BN) were obtained from XFNANO materials Tech Co. Thermoplastic polyurethane (TPU 1185A) was purchased from BASF Chemical Co. Ltd. Cell Counting Kit‐8 (CCK‐8) was purchased from BestBio Co. Ltd. L929 mouse fibroblasts cells was purchased from Procell Life Science & Technology Co. Ltd.

### Synthesis of MXene Nanosheets

MXene (Ti_3_C_2_T*_x_*) nanosheet was obtained through the same method as reported in the previous paper.^[^ ^14^ ^]^

### Preparation of MXene Nanosheets‐Coated Surface Microdomes‐Contained TPU/BN/MXene Film

First, the closely and uniformly arranged hexagonal monolayer array microstructure of the self‐assembled polystyrene (PS) microspheres on polydimethylsiloxane (PDMS) substrate could be obtained by the rubbing process. Then, the PDMS gel was coated on the self‐assembled PS microspheres‐based hexagonal monolayer array microstructure surface, and the negative concave PDMS template could be obtained by curing of PDMS gel at 60 °C and then sacrificing the PS microspheres‐based hexagonal monolayer array microstructure by immersing in toluene solution for 12 h. Subsequently, the surface microdomes‐contained TPU/BN film was obtained by coating TPU/BN‐contained DMF solution onto the PDMS template, curing, and peeling off. After treated with oxygen plasma for the surface microdomes‐contained TPU/BN film to increase the hydrophilicity of the template for further efficient MXene coating, the MXene nanosheets were coated on the surface of the surface microdomes‐contained TPU/BN film to obtain TPU/BN/MXene film with MXene nanosheets‐covered surface microdomes.

### Fabrication of Flexible Electronics

The interdigitated electrodes (15 mm × 10 mm) were obtained by screen‐printing the conductive silver paste onto the TPU/BN film with a commercial mask. Then, the MXene nanosheets‐coated surface microdomes‐contained TPU/BN/MXene film was assembled with the interdigitated electrode, which was connected to copper wires with the help of the conductive silver adhesive coating for further connecting to the circuit. The electronic skin was attached to the surface of the human skin with the help of medical tape. The pores of the flexible breathable porous wearable electronics were punched by an electric drill with a tungsten steel alloy drill bit (diameter at 0.2 mm) from Shenzhen Aoshi Weiye Electronics Co. Ltd.

### Machine Learning for Recognizing Different Tones

The dataset included signals for five different tones collected from different finger‐pressing sequence to seven sensors integrated on piano keyboards. Subsequently, the dataset was split into 80% for training and 20% for testing. Then the sample matrix was flattened, and a 1D‐CNN algorithm was employed to facilitate the learning process. The CNN model architecture comprised of three convolutional layers and two max pooling layers. Finally, the two fully connected layers were constructed for data classification. The Rectified linear units were employed to bring in nonlinearity. Additionally, the cross‐entropy loss function and the Adam optimizer were employed to enhance the network's performance. The model was trained for 100 epochs, and the classification accuracy and the cross‐entropy loss were used to assess the model's effectiveness in classifying tones.

### In Vitro Cytotoxicity Test

The TPU/BN films treated by UV sterilization were immersed in a cell culture medium at 37 °C for 24 h, and the solutions were filtered to obtain the extraction solution. Then, the L929 fibroblasts suspension was pipetted into a 24‐well plate at a density of 6 × 104 cells per well and cultured at 37 °C in a 5% CO_2_ atmosphere. After the culture medium was replaced with the extraction solutions, the cells were cultured at 37 °C in a 5% CO_2_ atmosphere for 24, 48, and 72 h, and CCK‐8 solution was added into the medium of each well and co‐cultured for 2 h. Finally, the cells were stained with Calcein‐AM for subsequent observation by the confocal laser scanning microscope.

### Characterization and Measurements

XRD and XPS were obtained from Rigaku D/Max 2500 and Thermo ESCALAB 250 respectively. The morphologies of the composites were obtained by scanning electron microscopy (Hitachi S‐4800), and transmission electron microscopy (Hitachi HT7700). The surface temperatures of the samples were recorded by the IR thermal (Fluke TiS75, Testo 875i) imager. The piezoresistive properties of the sensors were measured by an electrochemical workstation (CHI660E) and copper clamps. The volunteers signed the informed consent before human healthcare sensing without physical or psychological effect on volunteers’ skin during and after human healthcare sensing by coating flexible electronics on volunteers’ skin.

## Conflict of Interest

The authors declare no conflict of interest.

## Author Contributions

P.D. and Y.Y. contributed equally to this work. P.D. and Y.Y. did the investigation, resources, and data curation, and wrote the original draft. P.W. dealt with conceptualization, methodology, supervision, and Writing‐Review and Editing. M.X., Z.X., Y.L., C.H., G.X., and L.Z. dealt with Resources, Validation, and Software.

## Supporting information

Supporting Information

## Data Availability

The data that support the findings of this study are available from the corresponding author upon reasonable request.
