# Supplementary material for: A Flexible Skin Bionic Thermally Comfortable Wearable for Machine Learning‐Facilitated Ultrasensitive Sensing
Source: Adv Sci (Weinh). 2024 Jun 25;11(32):2401800. doi: 10.1002/advs.202401800 (PMC11348057; doi:10.1002/advs.202401800)
Supplement: Supplementary file 1 — Supporting Information [file ADVS-11-2401800-s001.pdf]

ADVANCED SCIENCE
Open Access

Supporting Information

for *Adv. Sci.*, DOI 10.1002/advs.202401800

A Flexible Skin Bionic Thermally Comfortable Wearable for Machine Learning-Facilitated Ultrasensitive Sensing

*Pengju Di, Yue Yuan, Mingyue Xiao, Zhishan Xu, Yicong Liu, Chenlin Huang, Guangyuan Xu, Liqun Zhang and Pengbo Wan**

Supporting Information

A Flexible Skin Bionic Thermally Comfortable Wearable for Machine Learning-Facilitated Ultrasensitive Sensing

Pengju Di,[#] *Yue Yuan,*[#] *Mingyue Xiao, Zhishan Xu, Yicong Liu, Chenlin Huang, Guangyuan Xu, Liqun Zhang, and Pengbo Wan**

P. J. Di, Y. Yuan, M. Y. Xiao, Z. S. Xu, C. L. Huang, Prof. L. Q. Zhang, Prof. P. B. Wan
College of Materials Science and Engineering, State Key Laboratory of Organic-Inorganic Composites, Beijing University of Chemical Technology, Beijing 100029, China
Email: pbwan@mail.buct.edu.cn

Y. C. Liu, Prof. G. Y. Xu
School of Artificial Intelligence, Beijing University of Posts and Telecommunications, Beijing 100876, China

[#]These authors contribute equally to this work.

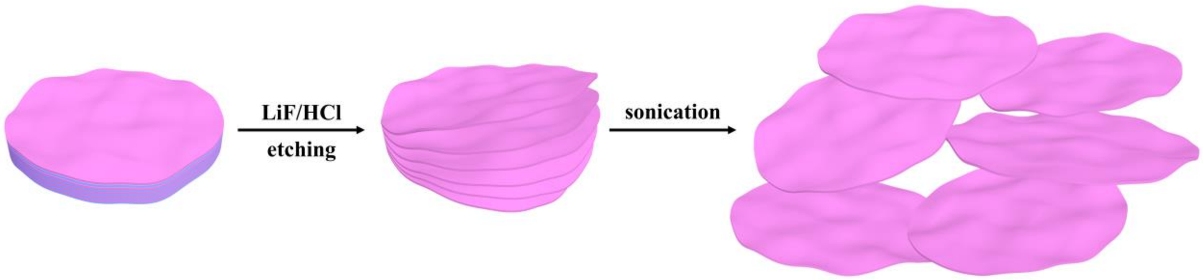

Figure S1. Scheme illustration for the synthesis of MXene nanosheets.

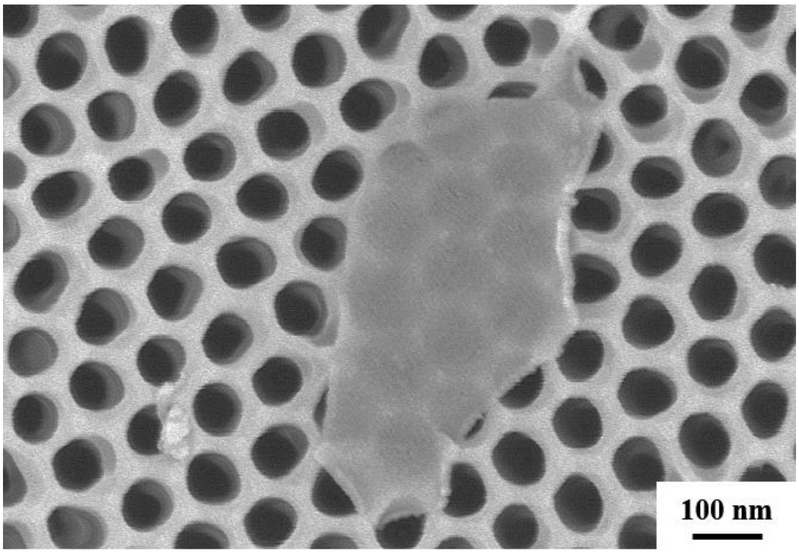

Figure S2. SEM image of MXene nanosheet.

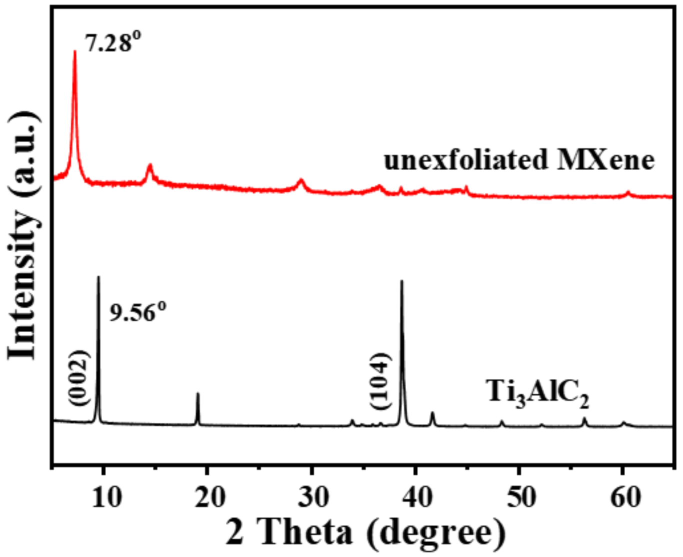

Figure S3. XRD patterns of Ti_3AlC_2 and unexfoliated MXene.

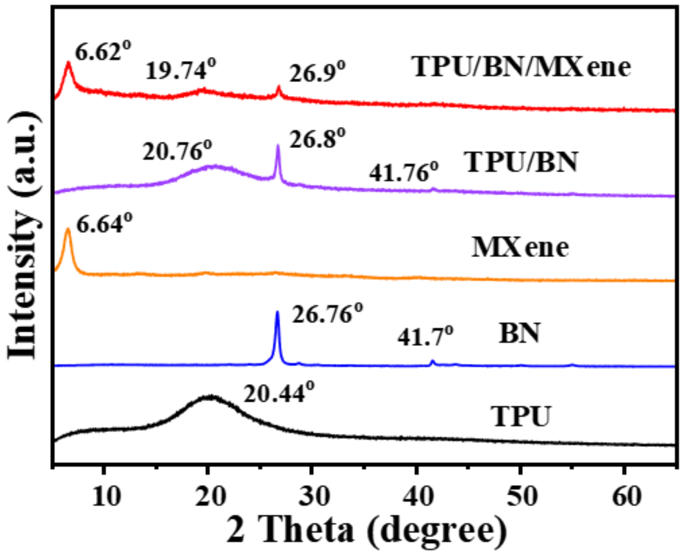

Figure S4. XRD patterns of TPU, BN, MXene, TPU/BN, TPU/BN/MXene.

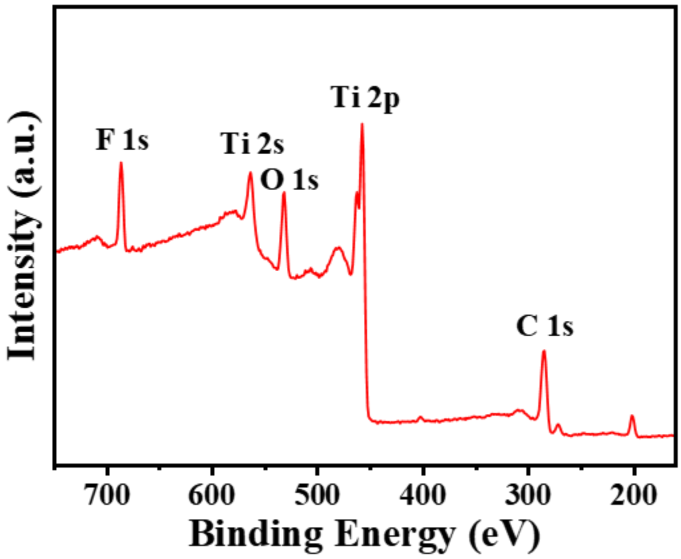

Figure S5. XPS pattern of the MXene nanosheet.

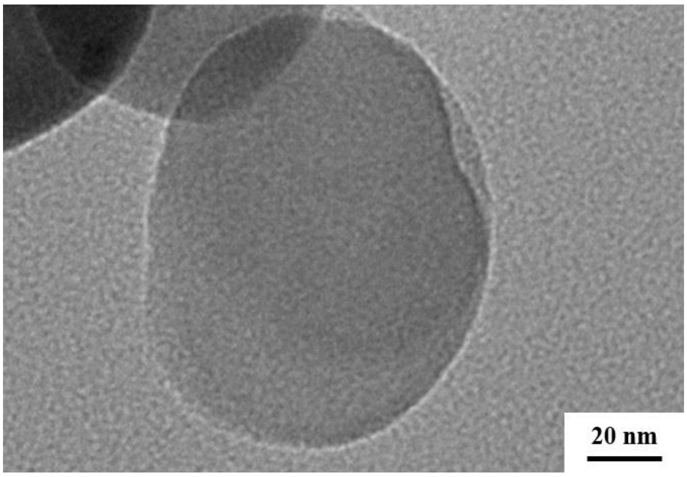

Figure S6. TEM image of the BN.

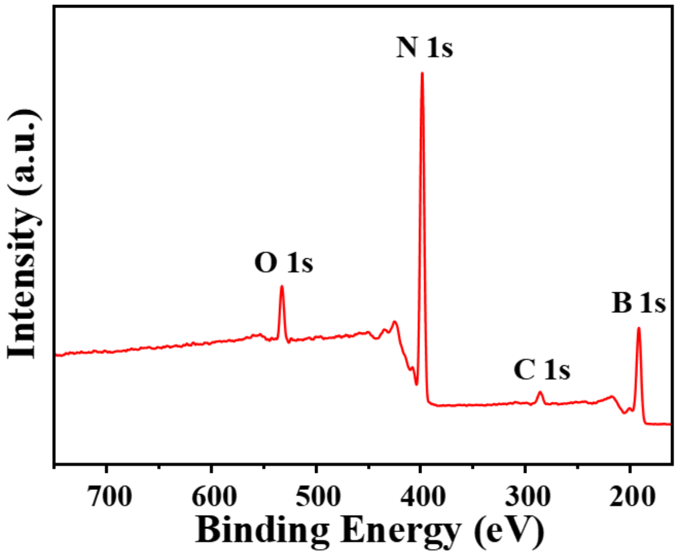

Figure S7. XPS spectra of BN.

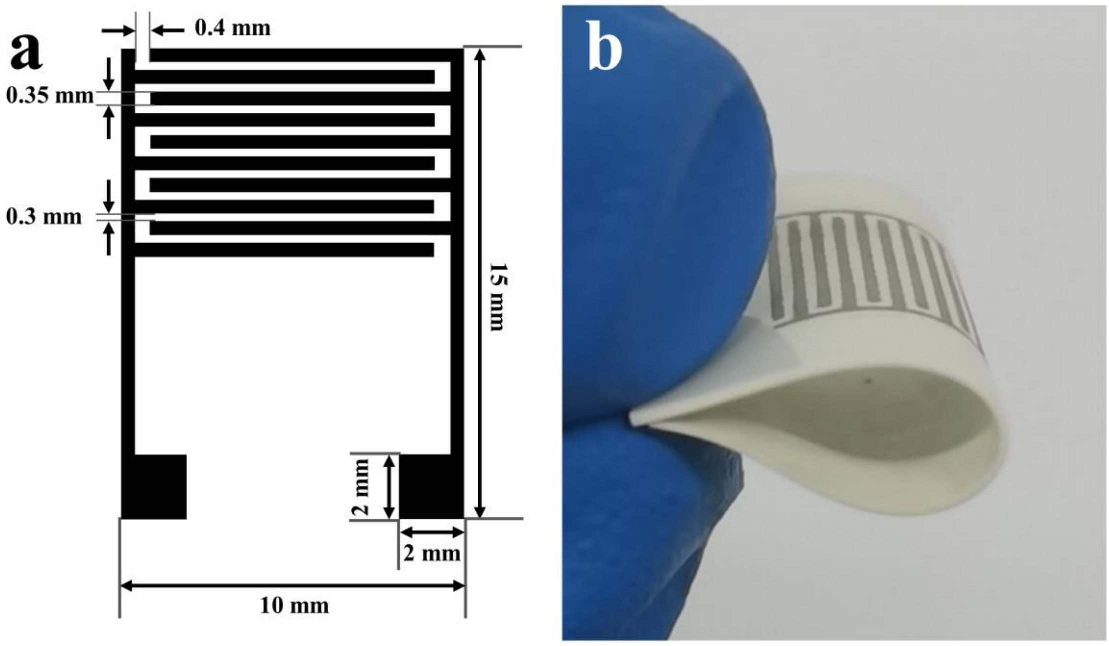

Figure S8. (a) The dimension and (b) the photograph of the TPU/BN/IE film.

a

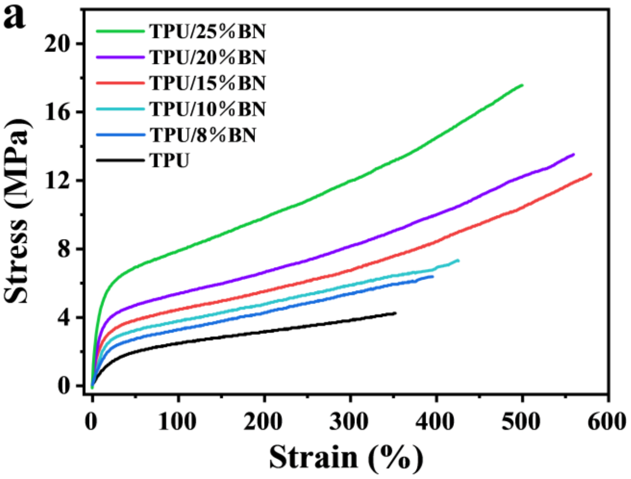

b

Materials	Elastic moduli
TPU/25%BN	24.9
TPU/20%BN	18.4
TPU/15%BN	13.6
TPU/10%BN	13.2
TPU/8%BN	6.8
TPU	3.9

Figure S9. (a) The tensile stress-strain curves of the TPU elastomers incorporated with various BN contents and (b) the corresponding variable elastic moduli.

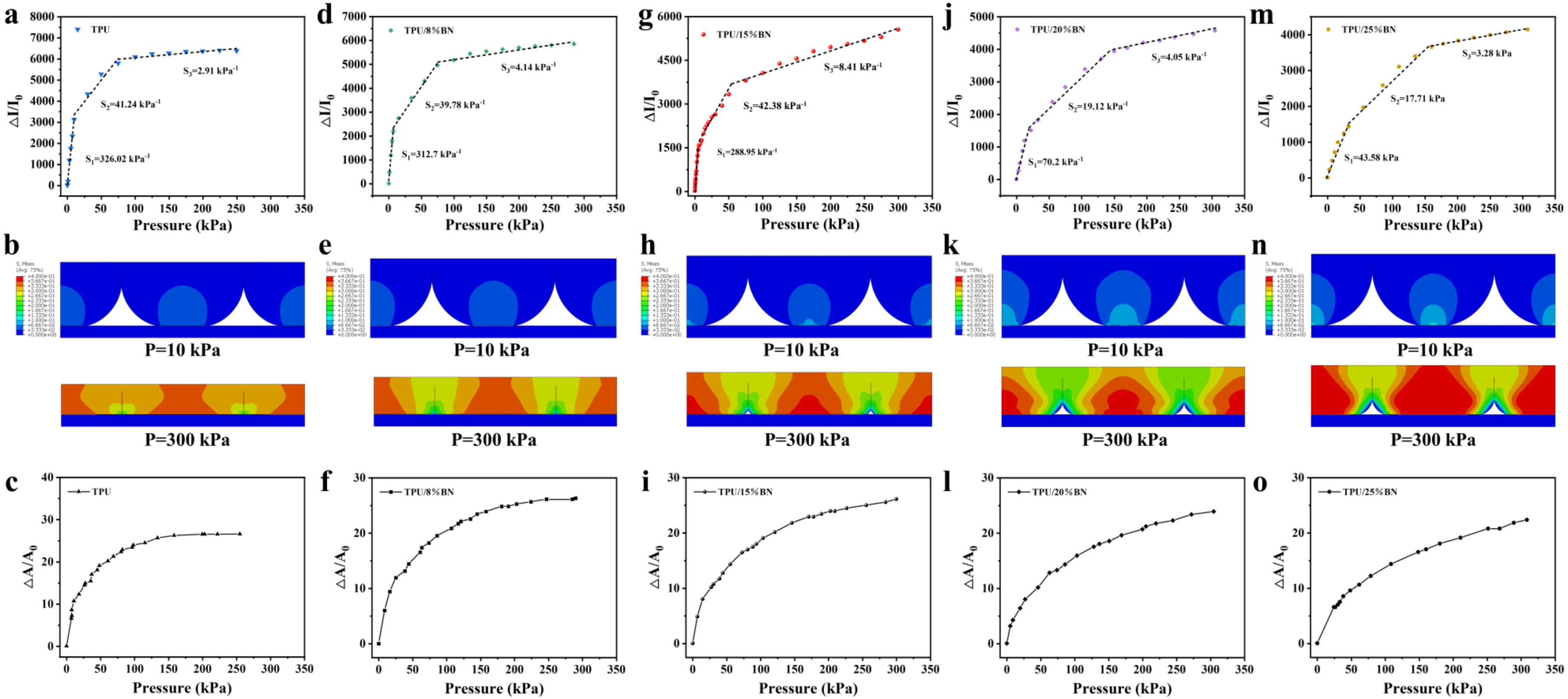

Figure S10. The sensing properties and the finite-element simulation results of the microdome array structures prepared with elastomer matrix of different elastic moduli. (a) The sensing capabilities of the skin bionic flexible electronic sensor made from pure TPU matrix with elastic moduli at ~3.9 MPa. (b) The stress distribution of the microdome array structures prepared from pure TPU matrix under the external pressures of 10 and 300 kPa respectively from the finite-element simulation (FEA). (c) FEA simulation results of the relative contact area change ($\Delta A/A_0$) from the microdome array microstructures prepared from pure TPU matrix and the contacted flat electrode under different external pressures loading. (d) The sensing capabilities of the skin bionic flexible electronic sensor made from TPU with 8 wt% BN content (TPU/8 wt%BN, elastic moduli at ~6.8 MPa). (e) The stress distribution of the microdome array structures prepared from TPU/8 wt%BN under the external pressures of 10 and 300 kPa respectively from the finite-element simulation (FEA). (f) FEA simulation results of the relative contact area change ($\Delta A/A_0$) from the microdome array microstructures prepared from TPU/8 wt%BN and the contacted flat electrode under different external pressures loading. (g) The sensing capabilities

of the skin bionic flexible electronic sensor made from TPU with 15 wt% BN content matrix (TPU/15 wt%BN, elastic moduli at ~13.6 MPa). (h) The stress distribution of the microdome array structures prepared from TPU with TPU/15 wt%BN under the external pressures of 10 and 300 kPa respectively from the finite-element simulation (FEA). (i) FEA simulation results of the relative contact area change ($\Delta A/A_0$) from the microdome array microstructures prepared from TPU/15 wt%BN matrix and the contacted flat electrode under different external pressures loading. (j) The sensing capabilities of the skin bionic flexible electronic sensor made from TPU with 20 wt% BN content matrix (TPU/20 wt%BN, elastic moduli at ~18.4 MPa). (k) The stress distribution of the microdome array structures prepared from TPU/20 wt%BN under the external pressures of 10 and 300 kPa respectively from the finite-element simulation (FEA). (l) FEA simulation results of the relative contact area change ($\Delta A/A_0$) from the microdome array microstructures prepared from TPU/20 wt%BN matrix and the contacted flat electrode under different external pressures loading. (m) The sensing capabilities of the skin bionic flexible electronic sensor made from TPU with 25 wt% BN content matrix (TPU/25 wt%BN, elastic moduli at ~24.9 MPa). (n) The stress distribution of the microdome array structures prepared from TPU/25 wt%BN under the external pressures of 10 and 300 kPa respectively from the finite-element simulation (FEA). (o) FEA simulation results of the relative contact area change ($\Delta A/A_0$) from the microdome array microstructures prepared from TPU/25 wt%BN matrix and the contacted flat electrode under different external pressures loading.

During the finite-element simulation process, the interdigitated electrode was simplified as a fixed rigid plate. The pressure was applied uniformly to the top of the model to compress the elastic microstructure downwards. To simulate the change of surface contact area, two contact modes were set as self-contact between each single dome in the microstructure model, and surface-to-surface contact between the microstructure model and interdigitated electrode model. The interfacial contact was assumed to be rough.

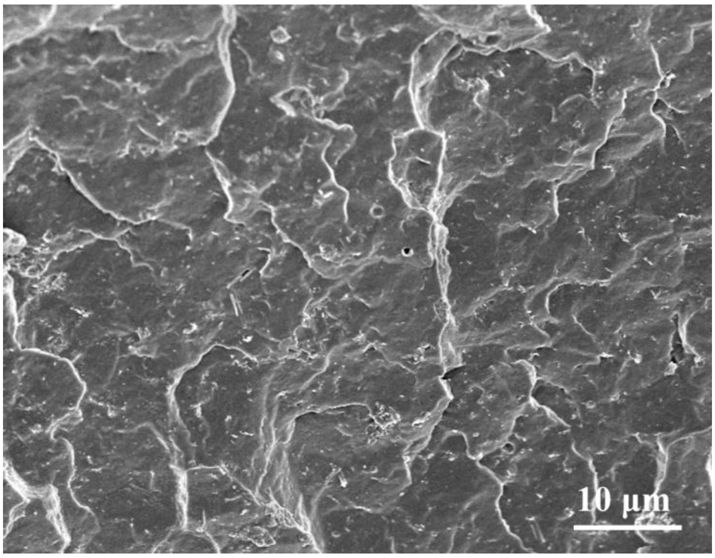

Figure S11. SEM image of thermal conductive BN network in TPU matrix.

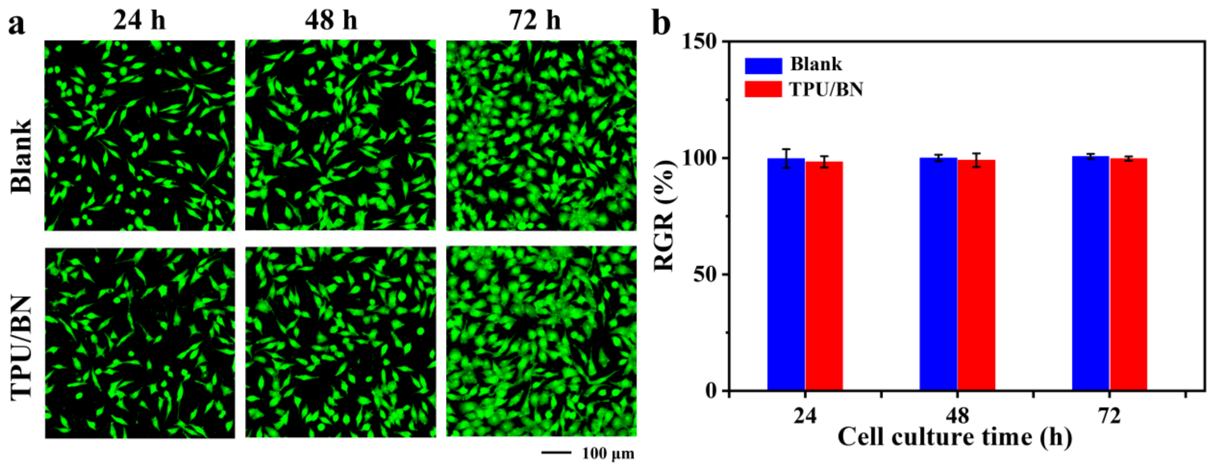

Figure S12. (a) The laser confocal microscope images of L929 cells after co-cultured with the extracts of TPU/BN composite and blank group respectively for 24 h, 48 h and 72 h. (b) The corresponding relative growth rate (RGR) values of L929 cells cultured with the extracts of TPU/BN composite and blank group respectively for 24 h, 48 h and 72 h respectively.

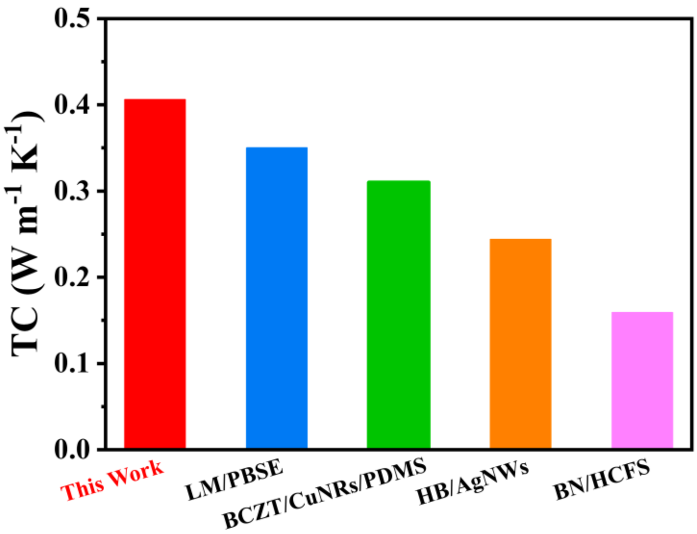

Materials	TC (W m^{-1} K^{-1})
TPU/BN	0.405
LM/PBSE	0.35
BCZT/CuNRs /PDMS	0.31
HB/AgNWs	0.244
BN/HCFS	0.159

Figure S13. The comparison of the thermal conductivity between TPU/BN composite with microdomes surface microstructure and other literatures[1-4].

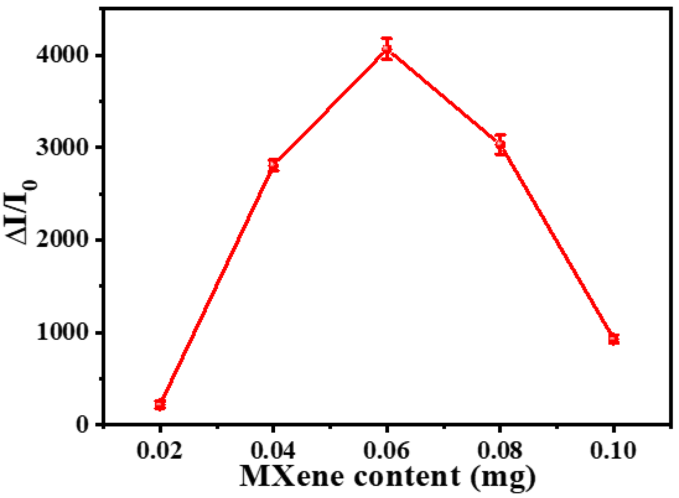

Figure S14. The sensing responses of the flexible electronics with the coating of different amounts of MXene nanosheets at 100 kPa.

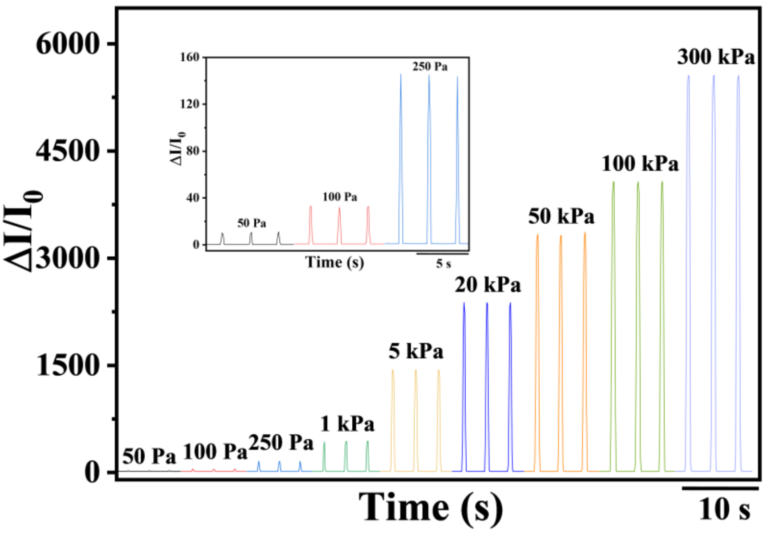

Figure S15. The sensing responses of the flexible electronics under various external pressures loading.

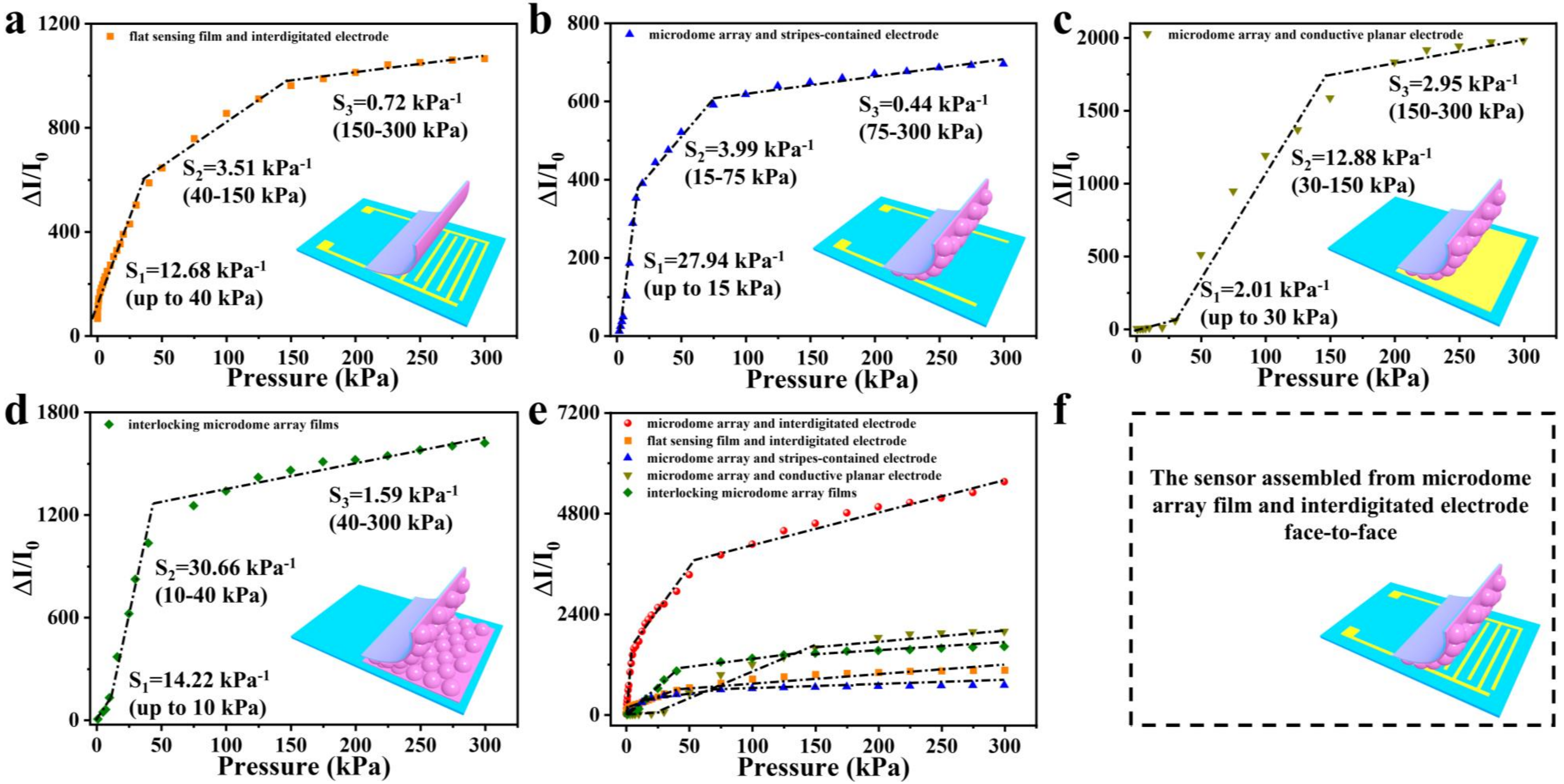

Figure S16. The perception performances of the sensors assembled from various sensing layers and different contact electrodes. (a) The perception performance of the sensor assembled from the flat TPU/BN/MXene film with MXene nanosheets coating and the interdigitated electrode-coated TPU/BN/IE film (Inset: the schematic of the corresponding as-assembled flexible electronics). (b) The perception performance of the sensor assembled from TPU/BN/MXene film with MXene nanosheets-covered surface microdomes and the two conducting stripes-contained electrode (Inset: the schematic of the corresponding as-assembled flexible electronics). (c) The perception performance of the sensor assembled from TPU/BN/MXene film with MXene nanosheets-covered surface microdomes and the conductive planar electrode (Inset: the schematic of the corresponding as-assembled sensor). (d) The perception performance of the sensor fabricated from the face-to-face assembly of two TPU/BN/MXene films with MXene

nanosheets-covered surface microdomes (Inset: the schematic of the corresponding as-assembled flexible electronics). (e) The perception performance comparison of the above four types of flexible electronics with the sensing sensitivity (the same sensing performance in Figure 3c) of (f) the sensor assembled from a TPU/BN/MXene film with the conductive MXene nanosheets-covered microdomes and an interdigitated electrode-covered TPU/BN film face-to-face.

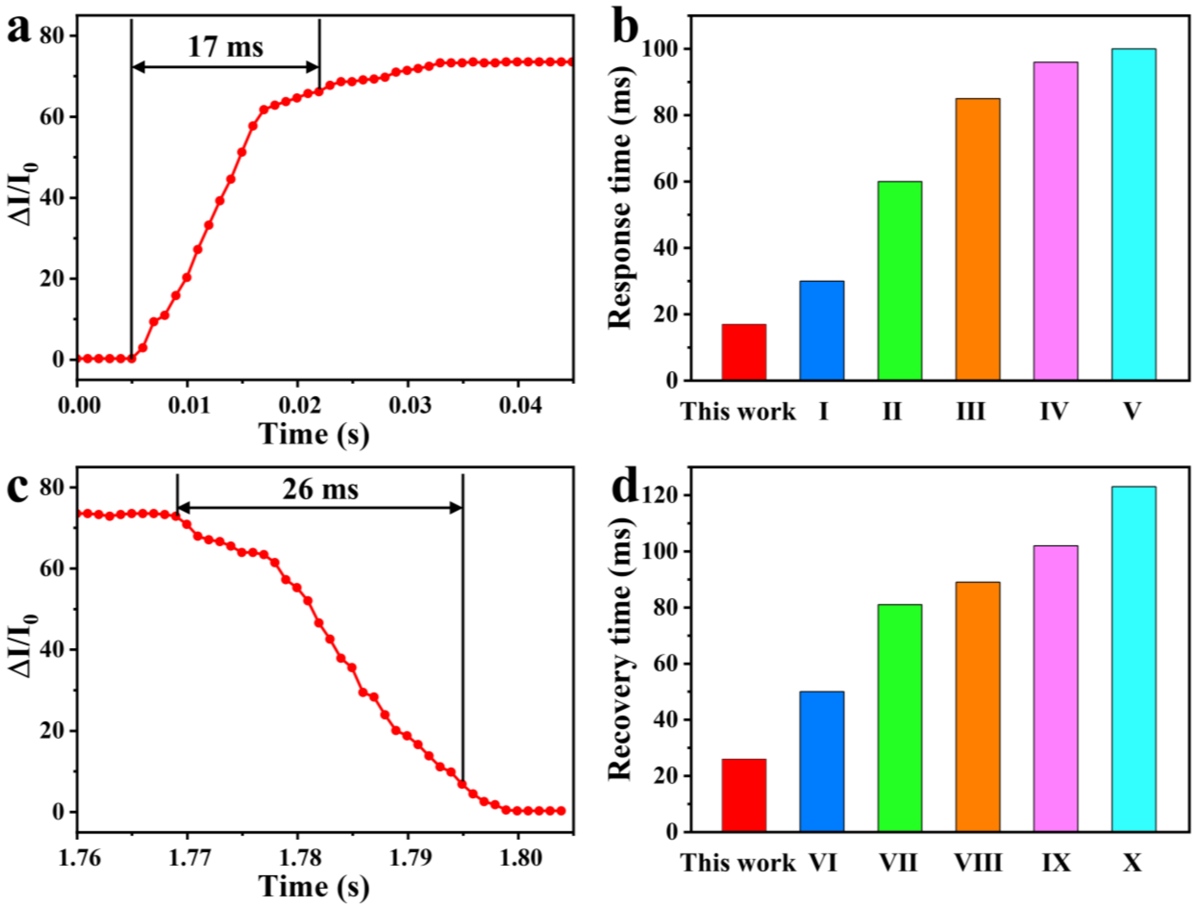

Figure S17. (a) The response time and (b) the corresponding response time comparison with other reports (I. PPy/PDMS (*Adv. Funct. Mater*. **2020**, *30*, 1907091); II. Carbon spheres/PDMS (*Nat. Commun*. **2020**, *11*, 3529); III. CNTs/PDMS (*Small* **2023**, *16*, 2208015); IV. CB/MWCNT/PDMS (*ACS Omega* **2022**, *7*, 44428); V. Au/PDMS (*ACS Appl. Mater. Interfaces* **2017**, *9*, 35968)). (c) The recovery time and (d) the corresponding recovery time comparison with other reports (VI. Au/PDMS/PET (*Adv. Healthc. Mater*. **2023**, *12*, 2301005); VII. MXene/AgNW/Nonwoven fabric (*Adv. Funct. Mater*. **2023**, *33*, 2214880); VIII. MXene/CNF (*Adv. Funct. Mater*. **2023**, *33*, 2211613); IX. Graphene/AgNFs/prslPDMS (*Nat. Commun*. **2023**, *14*, 1252); X. MXene/PEI (*Adv. Sci*. **2023**, *10*, 2205303)).

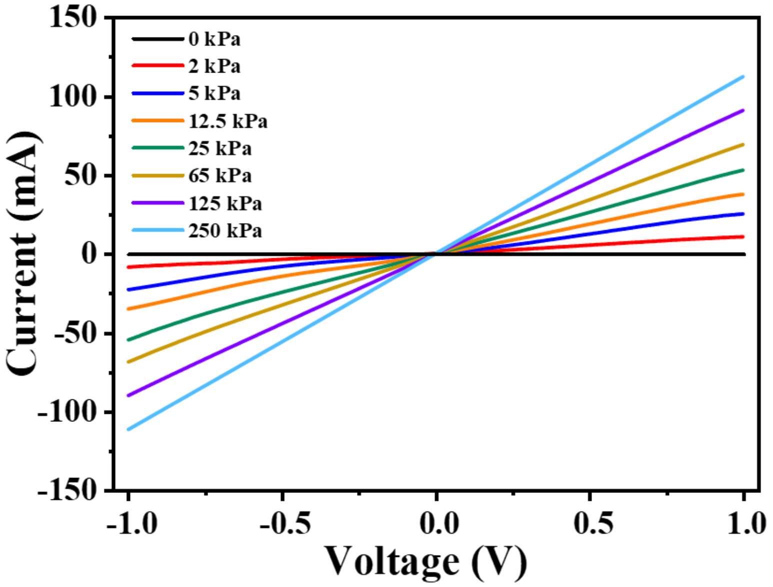

Figure S18. I-V curves of the flexible electronics under different pressures.

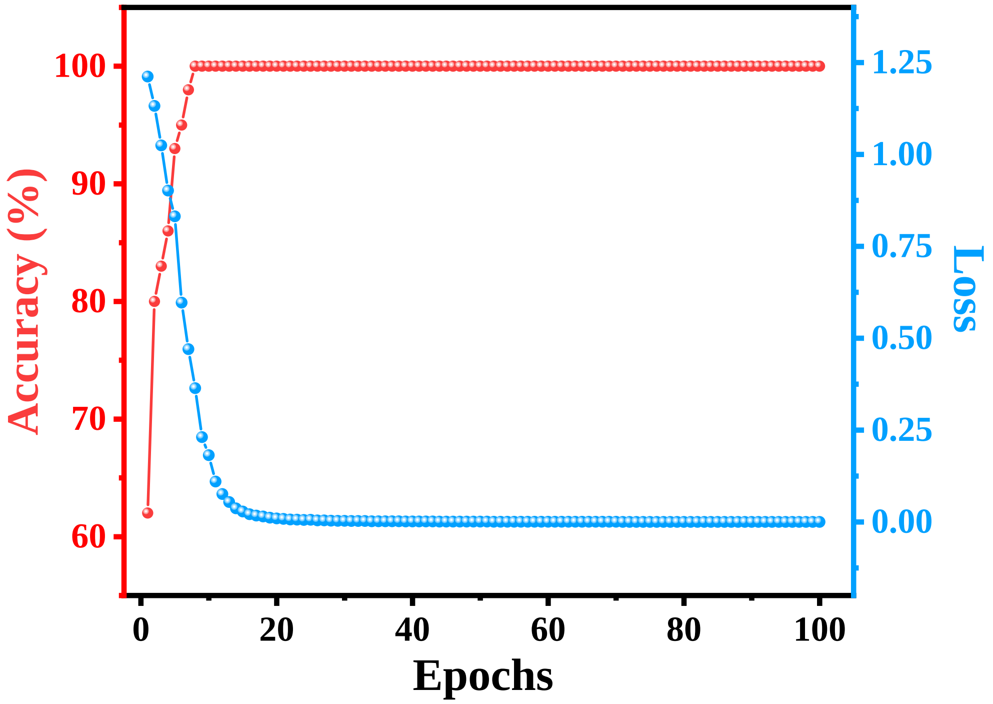

Figure S19. The classification accuracy and the loss function after 100 epochs training.

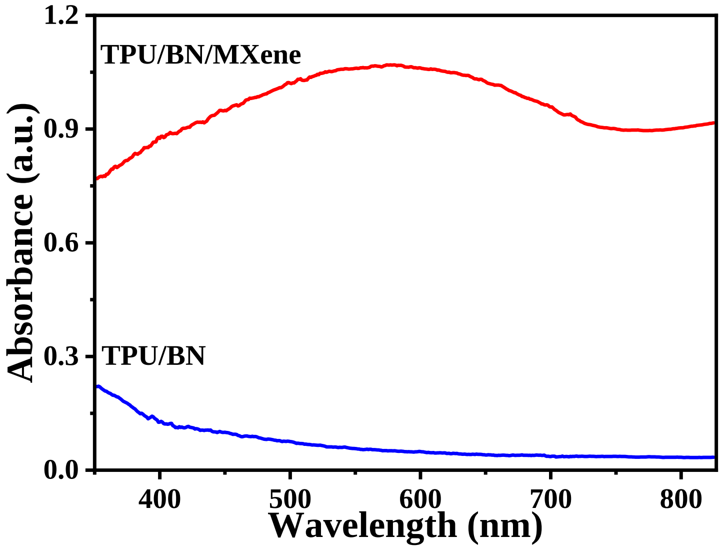

Figure S20. The normalized ultraviolet-visible-near infrared absorbance spectra (UV-Vis-NIR) of surface microdomes-contained TPU/BN film and MXene nanosheets-coated surface microdomes-contained TPU/BN/MXene film.

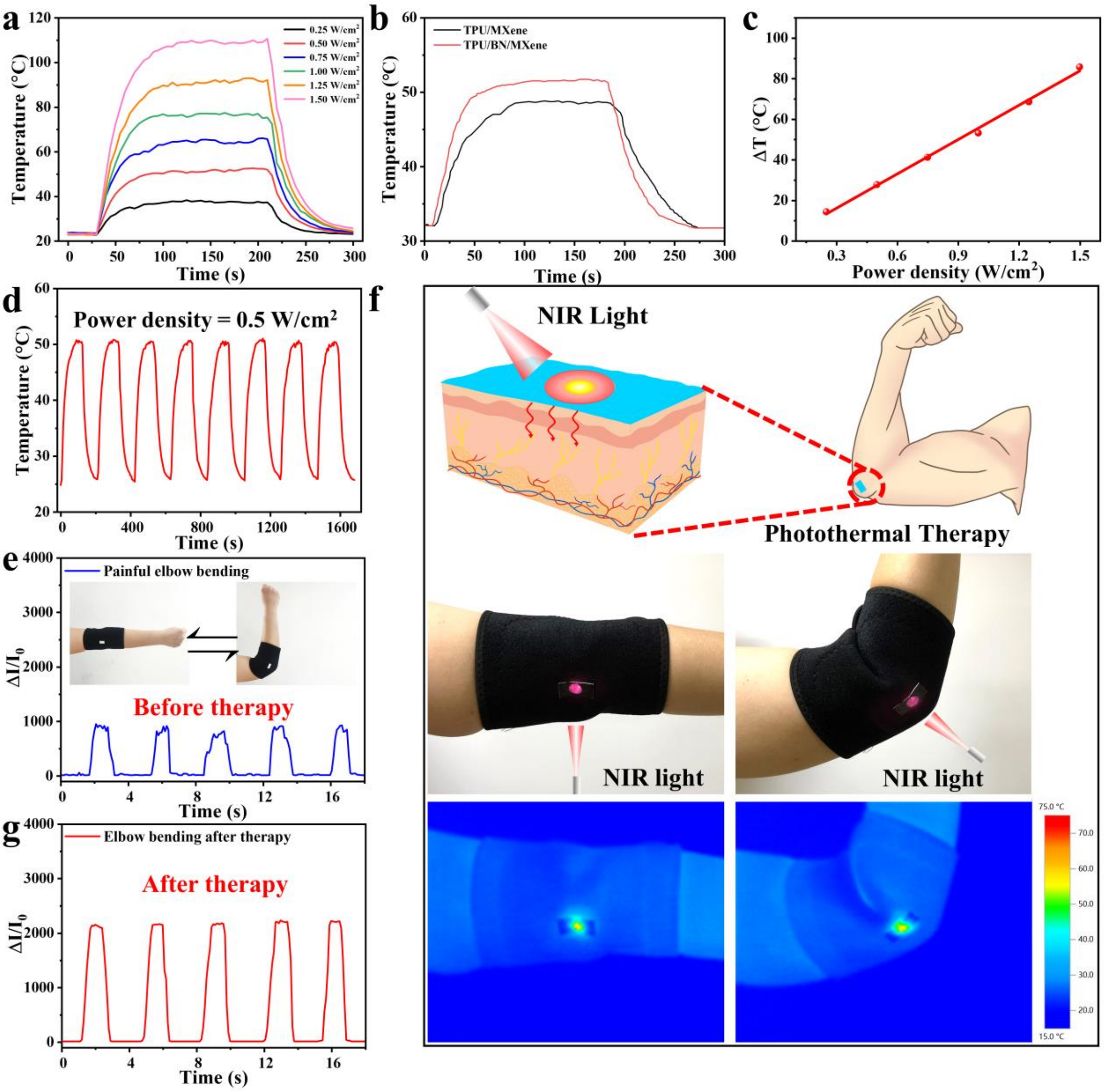

Figure S21. (a) Temperature−time curves of MXene nanosheets-coated surface microdomes-contained TPU/BN/MXene films under 808 nm NIR light irradiation at variable power densities. (b) The temperature−time curves of MXene nanosheets-coated surface microdomes-contained TPU/MXene film and MXene nanosheets-coated surface microdomes-contained TPU/BN/MXene film under 808 nm NIR light irradiation at the power density of 0.50 W/cm^{-2}. (c) The fitting curve between the temperature variation (from saturated temperature to experimental room temperature) of MXene nanosheets-coated surface microdomes-contained TPU/BN/MXene films and the different power densities. (d) The cycling temperature variation of MXene nanosheets-coated surface microdomes-contained TPU/BN/MXene films under 808 nm NIR light irradiation at 0.50 W/cm^{-2} power density. (e) The sensing responses of the flexible electronics to detect the bending of the painful elbow. (f) Schematic diagram of flexible electronic device for the photothermal therapy of elbow joint, and the photographs along with the corresponding infrared (IR) thermal images of elbow before and after bending when wearing the elbow band with the integrated MXene nanosheets-coated surface microdomes-contained TPU/BN/MXene film under 808 nm NIR light irradiation (0.50 W/cm^{-2}). (g) The sensing responses of the flexible electronics to detect the bending of the elbow after the photothermal therapy.

Table S1. The comparison for the sensing performance of the flexible electronics with that for the previously reported sensors.

Sensing materials	Maximum sensing range (kPa)	Highest sensitivity (kPa^{-1})	Number of cycles	Reference
TPU/BN/MXene	300	288.95	20000	This work
CNT/Textile	15	14.4	1000	5
PDMS/PPy/GR	50	2.01	10000	6
PPy/PDMS	32	120	1000	7
MXene/Au/PET	4.5	99.5	10000	8
rGO/Paper	20	17.2	300	9
MXene/rGO	3.5	22.56	10000	10
MXene/PDMS	15	151.4	10000	11
AgNW/GR/PANF	75	134	8000	12
C-MOF/PANIF/PU	60	158.26	15000	13
MXene/rGO/PS/PU	20.65	224	15000	14
MWCNT/PDMS	218	3.5	9000	15
AgNW/PU	204.7	6.25	2300	16
Thiohated Graphene/PET	200	8.36	500	17

[1] J. Huang, L. Hua, L. Song, J. Li, S. E, Z. Lu, Hydroxyethyl cellulose/boron nitride nanosheet films coated with silver nanowires for electronic packaging applications. *Mater. Today Sustain.* **2023**, *21*, 100307.

[2] D. Zong, X. Yin, J. Yu, W. Jiao, S. Zhang, B. Ding, Heat-conducting elastic ultrafine fiber sponges with boron nitride networks for noise reduction. *J. Colloid Interface Sci.* **2023**, *649*, 1023.

[3] C. Zhao, Y. Wang, L. Gao, Y. Xu, Z. Fan, X. Liu, Y. Ni, S. Xuan, H. Deng, X. Gong, High-performance liquid metal/polyborosiloxane elastomer toward thermally conductive applications. *ACS Appl. Mater. Interface* **2022**, *14*, 21564.

[4] X. Gao, M. Zheng, X. Yan, M. Zhu, Y. Hou, Ultrahigh current density and fatigue stability in flexible energy harvester by designing delivery paths. *Mater. Today Phys.* **2021**, *19*, 100424.

[5] M. Liu, X. Pu, C. Jiang, T. Liu, X. Huang, L. Chen, C. Du, J. Sun, W. Hu, Z. L. Wang, Large-area all-textile pressure sensors for monitoring human motion and physiological signals. *Adv. Mater.* **2017**, *29*, 1703700.

[6] H. Park, J. W. Kim, S. Y. Hong, G. Lee, D. S. Kim, J. h. Oh, S. W. Jin, Y. R. Jeong, S. Y. Oh, J. Y. Yun, J. S. Ha, Microporous polypyrrole-coated graphene foam for high-performance multifunctional sensors and flexible supercapacitors. *Adv. Funct. Mater.* **2018**, *28*, 1707013.

[7] S. Yu, L. Li, J. Wang, E. Liu, J. Zhao, F. Xu, Y. Cao, C. Lu, Light-boosting highly sensitive pressure sensors based on bioinspired multiscale surface structures. *Adv. Funct. Mater.* **2020**, *30*, 1907091.

[8] Y. Gao, C. Yan, H. Huang, T. Yang, G. Tian, D. Xiong, N. Chen, X. Chu, S. Zhong, W. Deng, Y. Fang, W. Yang, Microchannel-confined MXene based flexible piezoresistive multifunctional micro-force sensor. *Adv. Funct. Mater.* **2020**, *30*, 1909603.

[9] L.-Q. Tao, K.-N. Zhang, H. Tian, Y. Liu, D.-Y. Wang, Y.-Q. Chen, Y. Yang, T.-L. Ren, Graphene-paper pressure sensor for detecting human motions. *ACS Nano* **2017**, *11*, 8790.

[10] Y. Ma, Y. Yue, H. Zhang, F. Cheng, W. Zhao, J. Rao, S. Luo, J. Wang, X. Jiang, Z. Liu, N. Liu, Y. Gao, 3D synergistical MXene/reduced graphene oxide aerogel for a piezoresistive sensor. *ACS Nano* **2018**, *12*, 3209.

[11] Y. Cheng, Y. Ma, L. Li, M. Zhu, Y. Yue, W. Liu, L. Wang, S. Jia, C. Li, T. Qi, J. Wang, Y. Gao, Bioinspired microspines for a high-performance spray $Ti_3C_2T_x$ MXene-based piezoresistive sensor. *ACS Nano* **2020**, *14*, 2145.

[12] X. Li, Y. J. Fan, H. Y. Li, J. W. Cao, Y. C. Xiao, Y. Wang, F. Liang, H. L. Wang, Y. Jiang, Z. L. Wang, G. Zhu, Ultracomfortable hierarchical nanonetwork for highly sensitive pressure sensor. *ACS Nano* **2020,** *14*, 9605.

[13] Y. Wang, M. Chao, P. Wan, L. Zhang, A wearable breathable pressure sensor from metal-organic framework derived nanocomposites for highly sensitive broad-range healthcare monitoring. *Nano Energy* **2020**, *70*, 104560.

[14] L. Li, Y. Cheng, H. Cao, Z. Liang, Z. Liu, S. Yan, L. Li, S. Jia, J. Wang, Y. Gao, MXene/rGO/PS spheres multiple physical networks as high-performance pressure sensor. *Nano Energy* **2022**, *95*, 106986.

[15] T. Zhao, L. Yuan, T. Li, L. Chen, X. Li, J. Zhang, Pollen-shaped hierarchical structure for pressure sensors with high sensitivity in an ultrabroad linear response range. *ACS Appl. Mater. Interfaces* **2020,** *12*, 55362.

[16] G.-J. Zhu, P.-G. Ren, J. Wang, Q. Duan, F. Ren, W.-M. Xia, D.-X. Yan, A highly sensitive and broad-range pressure sensor based on polyurethane mesodome arrays embedded with silver nanowires. *ACS Appl. Mater. Interfaces* **2020**, *12*, 19988.

[17] L. Zhang, H. Li, X. Lai, T. Gao, J. Yang, X. Zeng, Thiolated graphene@polyester fabric-based multilayer piezoresistive pressure sensors for detecting human motion. *ACS Appl. Mater. Interfaces* **2018**, *10*, 41784.
